# Transgenic Expression of Soluble Human CD5 Enhances Experimentally-Induced Autoimmune and Anti-Tumoral Immune Responses

**DOI:** 10.1371/journal.pone.0084895

**Published:** 2014-01-15

**Authors:** Rafael Fenutría, Vanesa G. Martinez, Inês Simões, Jorge Postigo, Victor Gil, Mario Martínez-Florensa, Jordi Sintes, Rodrigo Naves, Kevin S. Cashman, José Alberola-Ila, Manel Ramos-Casals, Gloria Soldevila, Chander Raman, Jesús Merino, Ramón Merino, Pablo Engel, Francisco Lozano

**Affiliations:** 1 Institut d'Investigacions Biomédiques August Pi i Sunyer (IDIBAPS), Barcelona, Spain; 2 Departamento de Biología Molecular, Universidad de Cantabria, Santander, Spain; 3 Servei de Malalties Autoimmunes Sistémiques, Hospital Clínic de Barcelona, Barcelona, Spain; 4 Departament de Biologia Cel·lular, Immunologia i Neurociències, Universitat de Barcelona, Barcelona, Spain; 5 Department of Medicine, University of Alabama at Birmingham, Birmingham, Alabama, United States of America; 6 Department of Microbiology, University of Alabama at Birmingham, Birmingham, Alabama, United States of America; 7 Oklahoma Medical Research Foundation, Oklahoma City, Oklahoma, United States of America; 8 Departamento de Inmunología, Instituto de Investigaciones Biomédicas, Universidad Nacional Autónoma de México, Distrito Federal, México; 9 Instituto de Biomedicina y Biotecnología de Cantabria, Consejo Superior de Investigaciones Científicas-Universidad de Cantabria-SODERCAN, Santander, Spain; 10 Servei d'Immunologia, Hospital Clínic de Barcelona, Barcelona, Spain; MRC National Institute for Medical Research, United Kingdom

## Abstract

CD5 is a lymphoid-specific transmembrane glycoprotein constitutively expressed on thymocytes and mature T and B1a lymphocytes. Current data support the view that CD5 is a negative regulator of antigen-specific receptor-mediated signaling in these cells, and that this would likely be achieved through interaction with CD5 ligand/s (CD5L) of still undefined nature expressed on immune or accessory cells. To determine the functional consequence of loss of CD5/CD5L interaction *in vivo*, a new transgenic mouse line was generated (shCD5EμTg), expressing a circulating soluble form of human CD5 (shCD5) as a decoy to impair membrane-bound CD5 function. These shCD5EμTg mice showed an enhanced response to autologous antigens, as deduced from the presentation of more severe forms of experimentally inducible autoimmune disease (collagen-induced arthritis, CIA; and experimental autoimmune encephalitis, EAE), as well as an increased anti-tumoral response in non-orthotopic cancer models (B16 melanoma). This enhancement of the immune response was in agreement with the finding of significantly reduced proportions of spleen and lymph node Treg cells (CD4+CD25+FoxP3+), and of peritoneal IL-10-producing and CD5+ B cells, as well as an increased proportion of spleen NKT cells in shCD5EμTg mice. Similar changes in lymphocyte subpopulations were observed in wild-type mice following repeated administration of exogenous recombinant shCD5 protein. These data reveal the relevant role played by CD5/CD5L interactions on the homeostasis of some functionally relevant lymphocyte subpopulations and the modulation of immune responses to autologous antigens.

## Introduction

CD5 is a monomeric 67-kDa lymphocyte surface glycoprotein belonging to the scavenger receptor cysteine-rich superfamily (SRCR-SF) [1], [2]. This ancient and well conserved group of receptors includes both membrane-bound and/or secreted proteins involved in the development and the regulation of innate and specific immune responses [3], [4]. CD5 is expressed on thymocytes from early stages of their development, as well as on all mature peripheral T lymphocytes, and a subpopulation of peripheral B lymphocytes called B1a [5], with the highest expression levels found on T and B cells subsets with well-known regulatory/suppressor function (CD4+CD25+FoxP3+ Treg cells, and CD19+CD1d+IL-10+ Breg cells) [6]–[8]. On the surface of all these lymphoid cells, CD5 is physically associated to the T- and B-cell antigen-specific receptor (TCR and BCR, respectively) [9], and it is therefore well positioned to modulate the intensity of antigen-driven lymphocyte activation and differentiation signals.

Indeed, CD5 possesses a relatively large cytoplasmic domain devoid of enzymatic activity but harboring potential tyrosine and serine/threonine phosphorylation motifs compatible with a function in signal transduction [10], [11]. Engagement of CD5 may, however, result in different modulatory signals depending on the cell type analyzed and the maturation stage [12]. In mature T cells, CD5 was first considered as a co-stimulatory molecule for TCR receptor signaling [13], [14], but later studies showed a role for this molecule as a negative regulator of TCR signaling in thymocytes [15] and BCR signaling in B1a cells [16]. The fact that negative regulation does not occur in the absence of the CD5 cytoplasmic domain suggests that this effect depends on intracellular interactions mediated through signaling effector molecules [17]. It should be noted here that although several candidates have been proposed (CD72, gp35-40, IgV_H_, gp150, and CD5 itself), no CD5 ligand has been independently verified to date [3].

Although CD5 is a transmembrane molecule, a soluble form of human CD5 (shCD5) can be generated in activated T lymphocytes as a result of proteolytic cleavage [18]. Low levels of shCD5 (in the pico/nanomolar range) have been identified in the serum of healthy individuals, with discrete increased expression of this form reported in some autoimmune disorders such as rheumatoid arthritis [19], primary Sjögren's syndrome [20] and atopic dermatitis [21]. The functional relevance of sCD5 has not yet been elucidated; however, the fact that elevated levels of this protein have been found in certain autoimmune diseases suggests that sCD5 may be an indicator of chronic or exacerbated T cell activation, or alternatively, that it may play a role in the modulation of the immune response by interacting with the still uncharacterized endogenous CD5 ligand/s. Accordingly, affinity-purified shCD5 from pooled human sera was shown to bind a panel of different cell types of lymphoid (T and B), myeloid and epithelial origin. This cell binding pattern was identical to that obtained with a recombinant human sCD5 (rshCD5) molecule produced in a mammalian cell expression system and co-precipitating a new CD5L with a relative molecular mass of 150 kD [18]. Interestingly, rshCD5 induced a dose-dependent inhibition of polyclonal T-cell lympho-proliferative responses mediated by CD3, but not by phytohemagglutinin A (PHA) [22].

More recently it has been reported that both soluble and membrane-bound human CD5 molecules bind and sense the presence of pathogen-associated molecular patterns (PAMPs) of fungal origin (namely, beta-glucans) [23]. This pattern-recognition receptor (PRR) function is shared with a selected group of SRCR-SF members and has been hypothesized to be involved in both prevention of autoimmune phenomena and optimization of anti-microbial effector T cell responses during microbial aggression [24].

In an attempt to investigate the role of sCD5 in the homeostasis of immune cell populations, as well as during the course of the immune response, we generated a transgenic mouse line expressing a soluble form of human CD5 under the transcriptional control of the SV40 promoter and the immunoglobulin μ heavy chain enhancer (shCD5EμTg mice). Our assumption was that shCD5 would work as a decoy receptor, which would likely block the interactions between CD5 and its ligand/s (CD5L) whatever they are and wherever they are expressed. This is possible because we have observed cross-reactivity between human CD5 and murine CD5L, as has also been described for the highly homologous receptor CD6 [18]. Therefore, the study of these mice might shed light on the consequences of this interaction and, since the effects of the human protein can be tested, might serve as a test bed for future clinical use of shCD5. A similar approach has already been used for the study of other molecules, such as CTLA-4 [25] and VEGF-3 [26]. It is shown here that shCD5EμTg mice exhibit alterations in lymphocyte subpopulations with a regulatory/suppressive function, which can be reproduced in normal non-transgenic mice after repeated administration of exogenous rshCD5. Moreover, the expression of circulating shCD5 appears to be immunologically relevant, since shCD5EμTg mice showed increased severity of experimentally induced autoimmune disorders, and slower tumor growth compared with non-transgenic littermates. The latter anti-tumoral effects were also reproduced by repeated administration of purified rshCD5 to non-transgenic mice. Therefore, our data support a role for CD5/CD5L interactions in the homeostasis of some lymphocyte populations with regulatory and/or effector function, while highlighting the potential use of targeting CD5 as an immunomodulatory strategy in cancer therapy.

## Materials and Methods

### Animals

*In vivo* studies were carried out under protocols approved by the Ethics Committee for Animal Research of the University of Barcelona (permits number 498/12, 505/12 and 507/12), and were carried out in compliance with the International League of Associations for Rheumatology Guide for the Care and Use of Laboratory Animals, University of Cantabria, and the National Institutes of Health and University of Alabama at Birmingham Institutional Animal Care and Use Committee guidelines. All efforts were made to minimize animal suffering.

### Generation and genotyping of shCD5EμTg mice

The DNA sequence coding for the whole extracellular region of CD5 was amplified from the pHβAPRI-neo-CD5.P346^stop^ construct [18] and subcloned into XhoI-ApaI restricted pIgHSV40s vector which contained the SV40 promoter and the immunoglobulin μ heavy chain enhancer (Eμ). This vector, termed pIgHSV40shCD5, was injected into fertilized eggs from a CBAxC57Bl/6 mixed background. Founder mice were backcrossed for 10 generations into the C57Bl/6 background. Non-transgenic littermates were used in all experiments. Transgenic mice were identified by PCR analysis of genomic DNA samples from ear punch specimens in a GeneAmp PCR System 2700 termocycler (Applied Biosystems, USA). The cycling conditions were: 30 cycles of 5 min at 94°C, 1 min at 92°C, 1 min at 53°C, and 8 min at 72°C. The primers to detect the transgene were specific for the extracellular region of human CD5 (forward, 5′-GCTGTCCCAGTGCCACGAACTT-3′; reverse, 5′-GAAGCTCCTCTGTGTCCTCAT-3′). Internal control amplification primers (forward, 5′-TCACTCAAGGCAACCTTCCTGC-3′; reverse, 5′-CGACCTCATCTCTAACCATGAACAG-3′) specific for the invariant chain Ii of MHC class II (LIEX) were also included. The resulting PCR products (450 and 150 bp, respectively) were separated in a 2% agarose gel and visualized in a G:Box system (SynGene, UK).

### Production, purification and labeling of recombinant human proteins and mouse antibodies

The generation of expression constructs for recombinant soluble human CD5 and CD6 as well as their production and purification have been previously described [27], [28].

Mouse monoclonal antibodies (mAb) to human CD5, Cris-1 and Leu-1, were obtained from Dr. Ramon Vilella (Hospital Clínic of Barcelona) and the American Type Culture Collection (Manassas, VA), respectively, and purified by affinity chromatography using HiTrap Protein G columns (GE Healthcare).

Biotinylation of recombinant proteins and mAbs was performed with EZ-Link PEO-maleimide–activated biotin (Pierce) following the manufacturer's instructions.

### ELISA assays

The circulating levels of shCD5 were quantified by sandwich ELISA. 96-well plates (Nunc Maxisorb, Denmark) were coated overnight (o/n) at 4°C with 2 µg/mL of Cris-1 mAb as capture antibody, and then blocked for 1 h at 37°C with 2% bovine seroalbumin (BSA) in phosphate buffered saline (PBS). Neat mouse serum samples (100 µL) or serial dilutions of purified rshCD5, used as protein standard, were added to wells and incubated for 2 h at room temperature (RT). After washing three times with PBS 0.05% Tween 20 (PBS-T), 100 µL of 2 µg/mL of biotin-labeled Leu-1 mAb were added and incubated for 1 h at RT. Plates were washed again three times with PBS-T and incubated with horseradish peroxidase (HRP)-labeled streptavidin (Roche) for 30 min at RT. Color was developed in the plate using the ELISA Amplification System (Invitrogen, UK) and absorbance measured at 495 nm.

For detection of anti-human CD5 and CD6 mouse antibodies, ELISA plates were coated o/n at 4°C with 2 µg/mL of rshCD5 or rshCD6, respectively. After blocking with BSA, serially diluted mouse serum samples (100 µL) were added to the wells and incubated for 2 h at RT. After extensive washing with PBS-T, wells were incubated for 30 min at RT with HRP-labeled anti-mouse immunoglobulin antiserum (Sigma). Color was developed in the plate by addition of TMB substrate (BD Biosciences, USA) and absorbance measured at 450 nm.

Levels of TNP-specific antibodies of different isotypes were determined by using ELISA plates coated o/n with TNP_18_-BSA (3 µg/mL) in PBS. Once blocked with BSA, diluted 100 µL serum samples (1/500 for detection of IgM, and 1/10000 for all IgG isotypes) were added to the wells and incubated for 1 h at RT. Then, biotinylated sheep anti-mouse IgM, IgG1, IgG2a, IgG2b, IgG2c and IgG3 antisera (Jackson ImmunoResearch Laboratories, USA) were incubated for 1 h at RT. After three washes with PBS-T, HRP-labeled streptavidin was added and the plates developed with TMB.

### Immunoprecipitation and Western blot analysis of serum shCD5

Pooled sera from shCD5EμTg mice and non-transgenic littermates were sequentially precipitated to a final saturation of 20 and 70% ammonium sulfate. After extensive dialysis against PBS, the 70% protein precipitate was immunoprecipitated with Cris-1 mAb coupled CNBr-activated Sepharose (GE Healthcare) beads [18], [29]. Immunoprecipitates were separated on 10% agarose gels under non-reducing conditions and then Western blotted with biotin-labeled Leu-1 mAb and HRP-labeled streptavidin. Membranes were developed by chemiluminescence (ECL, Amersham Pharmacia).

### Immunization of mice with T-dependent (TD) and -independent (TI) antigens

To study the antibody response to TD and TI antigens, 7–8 week-old shCD5EμTg and non-transgenic littermates were used and immunized i.p. under different experimental conditions. For induction of anti-human CD5 and CD6 antibody responses, mice were primed with 25 µg of rshCD5 or rshCD6, in complete Freund's adjuvant (CFA, Sigma-Aldrich, UK) and boosted three weeks later with the same amount of protein in incomplete Freund's adjuvant (IFA). Two weeks after the boosting mice were bled and their sera analyzed by ELISA using rshCD5 or rshCD6 as the coating antigen (see above).

For induction of anti-trinitrophenyl (TNP) antibodies mice were given 50 µg of TNP_5_-KLH (as TD antigen) in 400 µl of PBS in CFA at day 0 and with vehicle (PBS) at day 14, TNP_0.3_-LPS (as type 1 TI antigen) or TNP_65_-Ficoll (as type 2 TI antigen) in 400 µl PBS. Antigen-conjugated TNP haptens were provided by Biosearch Technologies, Inc. (USA). Sera from immunized mice were collected at days 0, 7 and 14 (for TI response) or 0, 7, 14 and 21 (for TD response) days after the primary immunization and stored at −20°C until analysis.

### Flow cytometry analysis of mouse lymphocyte subpopulations

The following mAb were used to characterize lymphocyte subpopulations in mice: Fluorescein isothiocyanate (FITC)-labeled anti-mouse CD4 (eBioscience, US), B220 (eBioscience), BP-1 (eBioscience), IgD (eBioscience), CD21 (7G6, eBioscience) and CD62L (Tonbo Biosciences); Phycoerythrin (PE)-labeled anti-mouse CD23 (eBioscience), CD3γδ (eBioscience), B220 (eBioscience), NK1.1 (eBioscience), CD5 (eBioscience), CD43 (BD Biosciences), CD11b (M1/70, eBioscience), CD93 (AA 4.1), IgD (eBioscience), CD4 (L3T4), CD44 (BD Biosciences); PerCP-Cy5.5-labeled anti-mouse CD25 (BD Biosciences), CD5 (BioLegend) and CD19 (eBioscience); Allophycocyanin (APC)-labeled anti-mouse CD24 (M1/69), CD3ε (eBioscience), IgM (eBioscience) and CD25 (eBioscience); AlexaFluor 680-labeled anti-mouse IgM (eBioscience); AlexaFluor700 labeled anti-mouse CD8 (53-6.7); APC-eFluor 780-labeled anti-mouse B220 (BioLegend); anti-mouse IL-10 (JES5-16E3); Pacific blue-labeled anti-mouse B220 (BioLegend), GR1 (BD Biosciences) and CD11b (M1/70).

For staining, cell suspensions from spleen, lymph node and thymus were obtained using a cell strainer (50 µm). Splenocytes were depleted of erythrocytes by lysis with NH_4_Cl. Mouse peritoneal cells were obtained from peritoneal lavage with 4 mL PBS supplemented with 2% FCS and 0.01% sodium azide. Before surface staining with predetermined optimal concentrations of each mAb, cell samples (1×10^6^) were blocked by incubation with anti-CD16/32 2.4G2 mAb (Mouse BD Fc Block) for 30 min at 4°C.

For IL-10 intracellular staining, cells were stimulated with 50 ng/ml PMA, 500 ng/ml ionomycin (Sigma-Aldrich) and LPS (10 µg/ml, *Escherichia coli* serotype 0111:B4; Sigma-Aldrich) in the presence of Golgi-stop (monensin 2 µM; eBioscience) for 4 h at 37°C, in 96-well v-bottom plates. After surface staining with anti-B220, anti-CD5 and anti-CD1d antibodies, cells were fixed and permeabilized using a Cytofix/Cytoperm kit (BD Pharmingen), according to the manufacturer's instructions, and stained with PE-conjugated mouse anti-IL-10 mAb.

For FoxP3 intracellular staining, cells were stained with the PE-labeled anti-mouse/rat Treg Staining Kit (eBioscience), according to the manufacturer's instructions. Briefly, 1×10^6^ cells in suspension were stained with anti-CD4 and anti-CD25 antibodies and incubated with Fixation/Permeabilization Solution 18 h at 4°C. After this incubation, cells were washed twice with Permeabilization Buffer and incubated with anti-FoxP3 (Clone FJK-16s) 30 min. at 4°C.

Nine-color flow cytometry was performed on a BD FACSCanto flow cytometer (Becton Dickinson, US). Each analysis shown represents 500,000 events within the live lymphocyte gate. Flow cytometric profiles were analyzed using FlowJo software (Tree Star, USA).

### Collagen-induced arthritis (CIA) model

Type II bovine collagen (provided by Dr. M. Griffiths, University of Utah, Salt Lake City, USA) was dissolved at a concentration of 2 mg/mL in 0.05 M acetic acid and emulsified with CFA containing 4 mg/mL *Mycobacterium tuberculosis* (Chondrex, USA). For the induction of CIA, 8- to 10-week-old female non-transgenic and shCD5EμTg×(DBA _ B6)F1 hybrid mice were immunized once at the base of the tail with 150 µg of collagen II in a final volume of 150 µl. Clinical evaluation of arthritis severity was performed as described [30]. For radiological studies, mice were anesthetized by i.p. injection of a mixture containing: 50 mg/kg ketamine (Ketolar; Parke-Davis), 200 µg/kg atropine sulfate (Braun Medical, Spain), and 4 mg/kg diazepam (Roche, Spain). X-ray pictures were obtained using a CCX Rx ray source of 70 Kw with an exposure of 90 ms (Trophy Irix X-Ray System; Kodak, Spain). The radiological signal was digitalized with a Trophy RVG Digital Imagining system and analyzed using the Trophy Windows software. The severity of CIA was quantified radiologically with a graded scale according to the presence of five different radiological lesions (soft tissue swelling, juxtaarticular osteopenia due to alterations in bone density, joint space narrowing or disappearance, marginal erosions, and periosteal new bone formation). The extension of every individual lesion (local: affecting one digit or one joint in the carpus; diffuse: affecting two or more digits and/or two or more joints in the carpus) was graded from 0 to 1 as follows: 0, absence; 1/2, local; 1, diffuse. To clearly establish the radiological score in each paw, plain radiographs were assessed using a magnifying glass. Then, each paw was graded from 0 to 5, giving a maximum possible score of 20 for each mouse.

### Experimental autoimmune encephalomyelitis (EAE) model

C57Bl/6 non-transgenic and shCD5EμTg mice were immunized with a single s.c. injection of 150 µg of MOG_35–55_ peptide (BioSynthesis, USA) emulsified in CFA (Difco, supplemented with 400 µg/mL of Mycobacterium tuberculosis) on day 0, followed by one i.p. injection of 200 ng pertussis toxin (LIST Biologicals, USA) on day 0 and another on day 2 [31]. EAE symptoms were monitored daily for 30–35 days using a standard clinical score according to the following criteria: 0, no disease; 1, decreased tail tone; 2, hindlimb weakness or partial paralysis; 3, complete hindlimb paralysis; 4, forelimb and hindlimb paralysis; 5, moribund state [32]. Animals with a clinical score <2 as defined by loss of tail tone were considered free of clinical disease.

For histology, mouse brains were snap frozen and sectioned (10 µm) at peak disease scores, determined empirically for each mouse as the fifth day after initial disease signs (score >0). Tissue was stained with hematoxylin and eosin to detect CNS inflammatory infiltrates, or solochrome cyanin to assess myelination.

### *In vivo* tumor growth assays

For tumor studies, B16 melanoma cells, which are of murine C57Bl/6 origin, were used. For *in vivo* tumor growth, 5×10^4^ cells were injected s.c. on the mid-dorsum with a 23-gauge needle. Tumors were measured every other day with a Vernier calliper, and the diameter in mm of the length and width of the tumors averaged. Chemotherapy treatment, when used, was administered at day 3 following tumor cell injection and consisted of a single dose of 0.5 mg/kg vincristine and 3.3 mg/kg doxorubicin, injected i.p. For experimental metastasis assays, 1×10^5^ cells were injected i.v. After 15 days, mice were sacrificed, their lungs excised and the number of metastases on the lung surface counted. For both types of assays, results are expressed as mean ± standard error of mean (SEM).

### Statistical analysis

Statistical significance of differences between result groups was determined using Student's *t* test, unless stated otherwise. In all experiments, differences were considered statistically significant when *p* was<0.05.

## Results

### Generation and characterization of shCD5EμTg mice

In an attempt to investigate the functional relevance of the CD5/CD5L interactions *in vivo*, a new line of transgenic mice (shCD5EμTg) was generated, which expresses a soluble form of human CD5 (shCD5). This was done by taking advantage of interspecies conservation of the receptor-ligand interactions mediated by CD5, in a similar way to that reported for the highly homologous receptor CD6 [33]. Accordingly, both biotin-labeled shCD5 and shCD6 bound to activated human or mouse lymphocytes to a similar extent, as illustrated by **Figure S1**.

**Figure 1:**
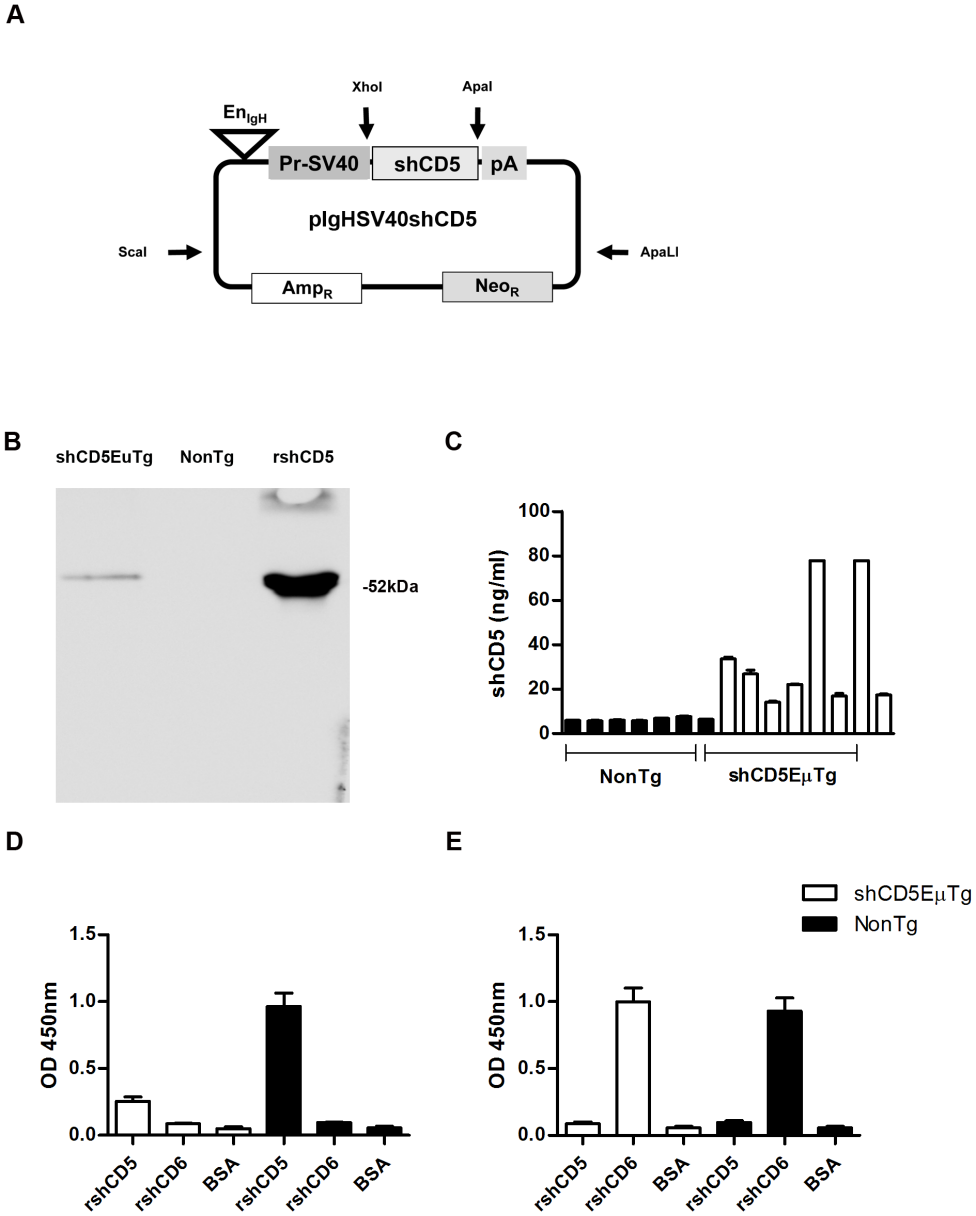
Generation and characterization of human soluble hCD5 transgenic mice (shCD5EμTg). **A**) Structure of the pIgHSV40shCD5 targeting vector. The cDNA sequence for shCD5 was inserted as *Xho*I-*Apa*I in between the promoter and polyA sequence of the pIg vector and then isolated as ScaI-ApaLI fragment for oocyte injections. **B**) Western blot analysis of human CD5 immunoprecipitates from transgenic (shCD5EμTg) and non-transgenic mice (NonTg) sera. The anti-CD5 Cris-1 mAb coupled with CNBr-activated Sepharose beads was used as immunoprecipitating agent. Western blotting was then carried out using the Leu-1 mAb plus HRP-labeled anti-mouse Ig. As a positive control, purified rshCD5 (50 ng) was included. **C**) ELISA detection of shCD5 in sera from shCD5EμTg mice. Sera from transgenic (shCD5EμTg, white) and non-transgenic littermates (NonTg, black) mice were analyzed by sandwich ELISA using Cris-1 and biotin-labeled Leu-1 as capture and developing mAbs, respectively. A standard rshCD5 curve was also analyzed in parallel to quantify results. **D–E**) shCD5EμTg mice are tolerant to exogenously administered rshCD5 but not rshCD6. shCD5EμTg mice and wild-type littermates were immunized twice with rshCD5 (**D**) or rshCD6 (**E**) (25 µg) in Freund's adjuvant (complete and incomplete, sequentially) within a 3-week interval. Mouse sera were collected 2 weeks after the boosting and added to ELISA plates coated with purified rshCD5, rshCD6 or BSA, then developed with a HRP-labeled anti-mouse IgG. Values represent the mean OD 450 nm values ± SD obtained in triplicate determinations for each sample (five mice per group). The total concentration of IgG was similar for both groups.

The shCD5EμTg mice were engineered to express the whole extracellular region of human CD5 (from initiation Met to Pro346) under the transcriptional control of the SV40 promoter and the B-cell specific Eμ enhancer (**Fig. 1A**). The transgene was injected into fertilized eggs of CBAxC57Bl/6 mixed background and the founder mice were backcrossed into the C57Bl/6 background. Transgenic mice were identified with a specific PCR for the extracellular region of human CD5 (**Figure S2**) and the transgenic expression of shCD5 mouse serum was confirmed by immunochemical studies. Thus, pooled sera from shCD5EμTg mice or non-transgenic littermates were immunoprecipitated with an anti-human CD5 mAb (Cris-1) and analyzed by Western blotting with a second anti-human CD5 mAb (Leu-1). As illustrated by **Figure 1B**, a 52-kDa band was detected in shCD5EμTg mice sera, which was absent from that of non-transgenic littermates. The serum levels of shCD5 expressed by shCD5EμTg mice were further analyzed by sandwich ELISA and were shown to be in the ∼10–100 ng/mL range (**Fig. 1C**).

Further confirmation on the transgenic expression of shCD5 was obtained from the demonstration that shCD5EμTg mice were tolerant to exogenously administered purified rshCD5, but not to rshCD6. To evaluate this, the titers of anti-shCD5 or anti-shCD6 antibodies in shCD5EμTg mice immunized with two consecutive doses (25 µg each) of either rshCD5 or rshCD6 were analyzed by ELISA two weeks after the second immunization. As shown in **Figure 1D**, sera from shCD5EμTg mice immunized with rshCD5 showed very low antibody levels against rshCD5, compared with those of non-transgenic mice sera. The specificity of the anti-CD5 antibody response was demonstrated by the lack of detection of antibodies reactive to both structurally similar (rshCD6) and unrelated (BSA) proteins. In contrast, when immunized with rshCD6 both shCD5EμTg mice and non-transgenic mice showed similarly high and specific titers of anti-shCD6 antibodies, which did not cross-react with related and unrelated protein controls (**Fig. 1E**). This indicates that shCD5EμTg mice are tolerant to human shCD5 but not to a highly homologous human protein such as shCD6.

### shCD5EμTg mice exhibit normal proportions of major T and B cell subpopulations and developmental stages

**Figure 2:**
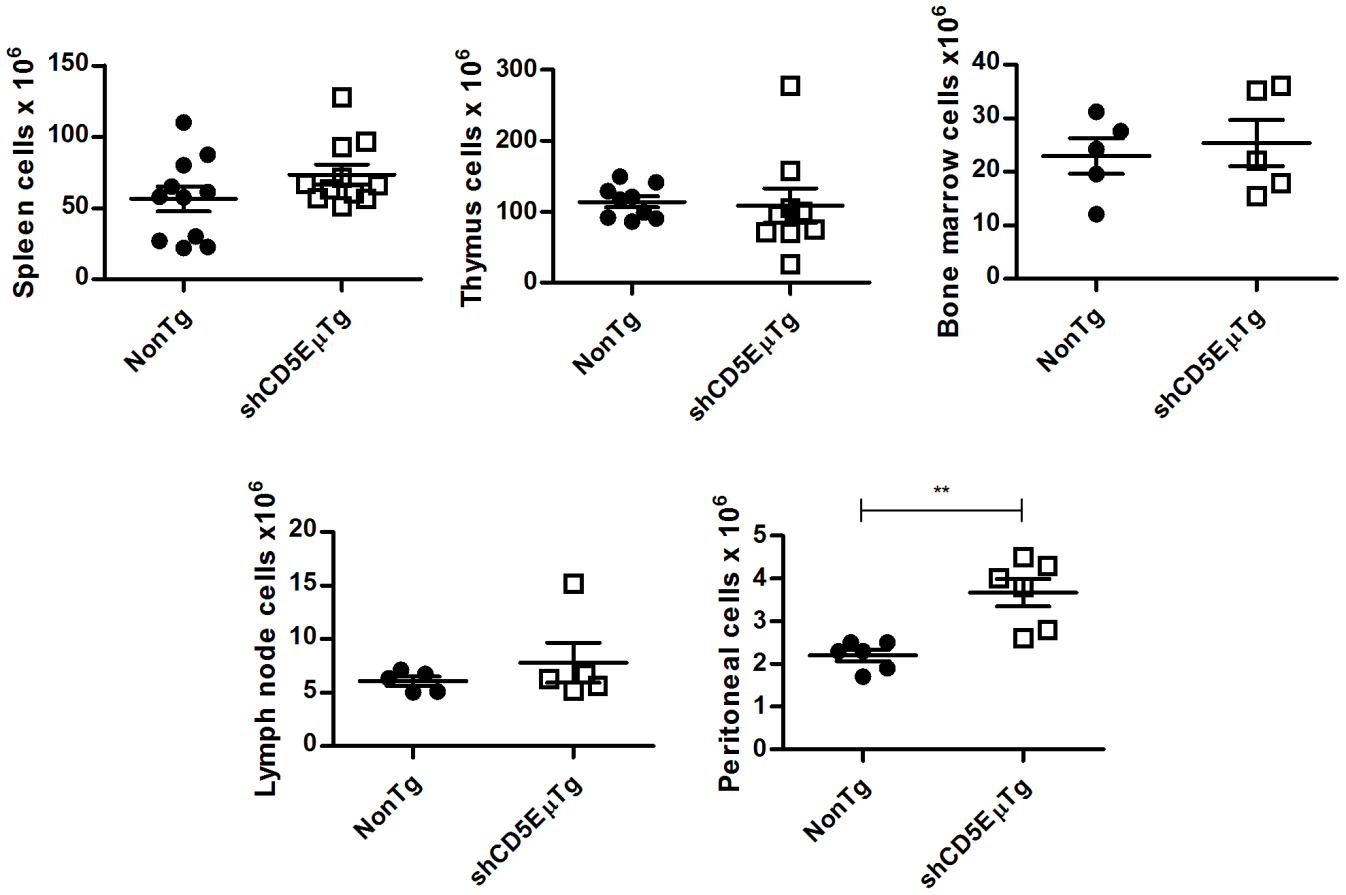
Analysis of total cell numbers in primary and secondary lymphoid organs from shCD5EμTg mice. Total number of mononuclear cells in spleen, thymus, bone marrow, lymph node and peritoneal lavage samples was measured by flow cytometry using forward and side scatter for gating. ** p = 0.0018.

To investigate the phenotype of shCD5EμTg mice regarding lymphocyte development and homeostasis, the total cell numbers and percentages of major lymphocyte subpopulations were analyzed in the thymus, lymph node, bone marrow, peritoneum and spleen. As shown in **Figure 2**, total cell numbers were not significantly different in the thymus, bone marrow or lymph node of shCD5EμTg mice compared with non-transgenic littermates, although the number of cells in peritoneum was significantly higher in shCD5EμTg mice; this increase could not be ascribed to any single population, as relative percentages of different cell subpopulations remained unchanged (data not shown). A higher number of cells was also observed in the spleen of shCD5EμTg mice although this did not reach statistical significance.

Further analysis of major T cell subsets (CD4+ and CD8+) from secondary lymphoid organs (lymph nodes and spleen) did not detect any gross abnormalities in lymphocyte subpopulation percentages of shCD5EμTg mice compared with non-transgenic littermates (**Fig. S3A and B**). A similar scenario was observed in thymus, where none of the different T cell developmental stages (DN1–DN4, DP, SP CD4+, SP CD8+) appeared to be affected (**Fig. S3C and D**).

**Figure 3:**
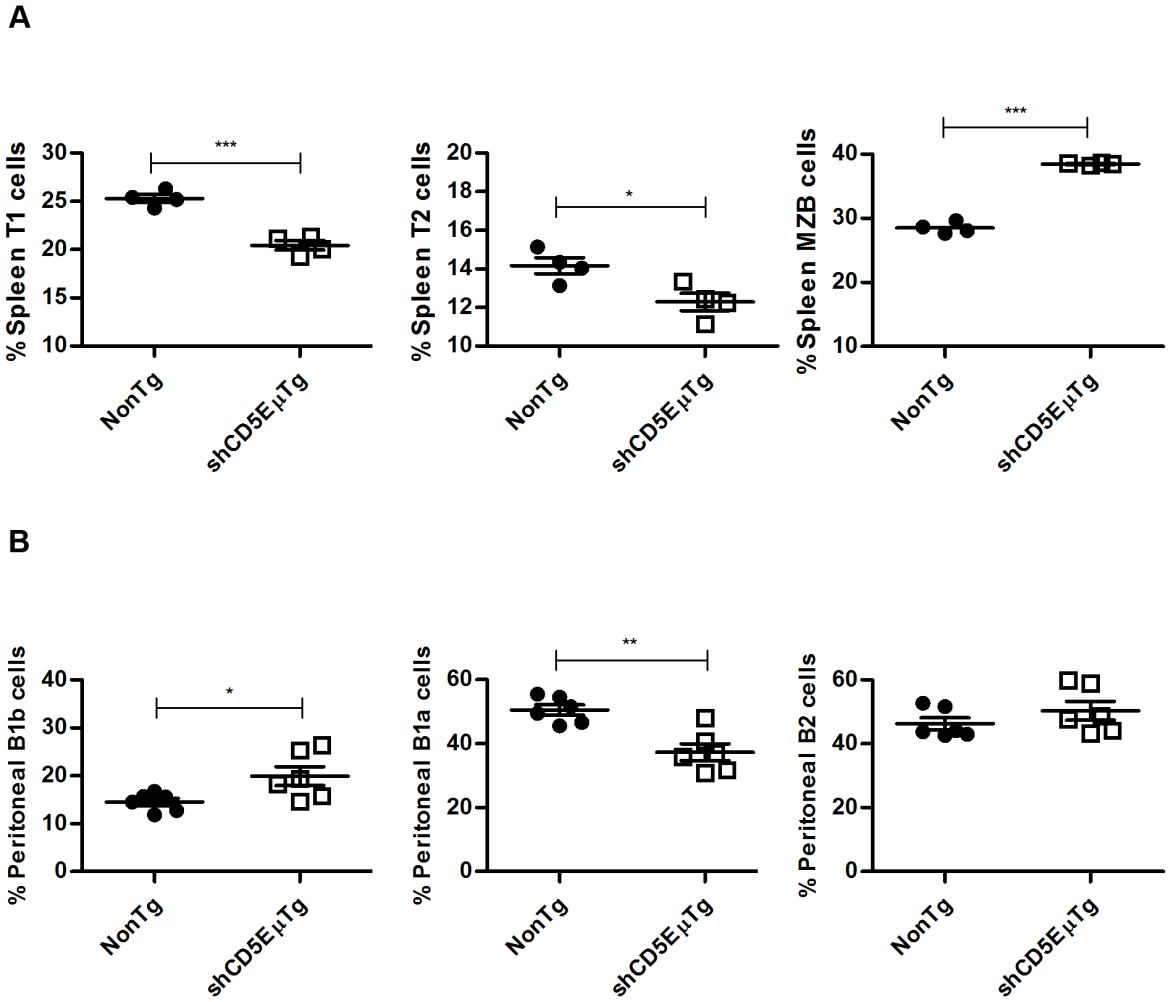
Flow cytometry analysis of B lymphocyte subpopulations in shCD5EμTg mice. A) To analyze peripheral B cell subsets from spleens of shCD5 transgenic mice and non-transgenic littermates, we used a gating strategy adapted from Cariappa *et al* [45]. Gated on IgM^high^IgD^low^, transitional 1 (T1), transitional 2 (T2) and marginal zone B cells can be distinguished based on their differential expression of the surface markers CD23 and CD93/AA4. *** p≤0.0003 * p = 0.0221. B) Cells obtained from peritoneal lavage samples were stained to distinguish B1a cells (B220+IgM^high^IgD^low^CD5+), B1b cells (B220+IgM^high^IgD^low^CD5−) and B2 cells (B220+IgM^high^IgD^high^CD5−) in shCD5 transgenic mice and non-transgenic littermates. * p = 0.0294 ** p = 0.0016.

B lymphocyte subsets from peripheral (spleen and peritoneum) and central (bone marrow) organs were also analyzed. As shown in **Figure 3A**, a significant decrease in the percentage of the transitional 1 (T1, IgM^hi^ IgD^lo^ CD93/AA4^+^ CD23^−^) and 2 (T2, IgM^hi^ IgD^lo^ CD93/AA4^+^ CD23^+^) cells as well as an increase in marginal zone B cells (MZB, IgM^hi^ IgD^lo^ CD93/AA4^lo^ CD23^−^) were detected in the spleen of shCD5EμTg mice compared with non-transgenic littermates. No significant differences were observed between shCD5EμTg and non-transgenic mice regarding the transitional 3 (T3, IgM^lo^ IgD^hi^ CD93/AA4^+^ CD21^+^), follicular I (FOLI, IgM^lo^ IgD^hi^ CD93/AA4^lo^ CD21^+^), follicular II (FOLII, IgM^hi^ IgD^hi^ CD93/AA4^+^ CD21/35^lo^) and MZ precursor (MZP, IgM^hi^ IgD^hi^ CD93/AA4^+^ CD21/35^hi^) cells (**Figs. S4 and 5A**). In a similar way to what was observed with T cell development, we did not find any major differences in B cell developmental stages in the bone marrow (Pro/pre-B, early pro-B, pro-B and pre-B cells) (**Fig. S6**).

However, as shown in **Figure 3B** **and Figure S5B**, analysis of peritoneal B cells showed a significant decrease in the percentage of B1a cells (B220+ IgM^hi^ IgD^lo^ CD5+) and a smaller, albeit significant, increase in the percentage of B1b cells (B220+ IgM^hi^ IgD^lo^ CD5−). No differences were observed in mature B2 cells (B220+ IgM^hi^ IgD^hi^ CD5−).

Taken together, phenotypical analysis of shCD5EμTg mice indicates that expression of shCD5 does not induce gross alterations on T and B cell development, and that the only abnormalities observed affect certain B cell subsets from spleen (T1, T2, MZB) and peritoneum (B1a, B1b).

### shCD5EμTg mice display reduced proportions of B and T lymphocyte subsets with regulatory/suppressor function

**Figure 4:**
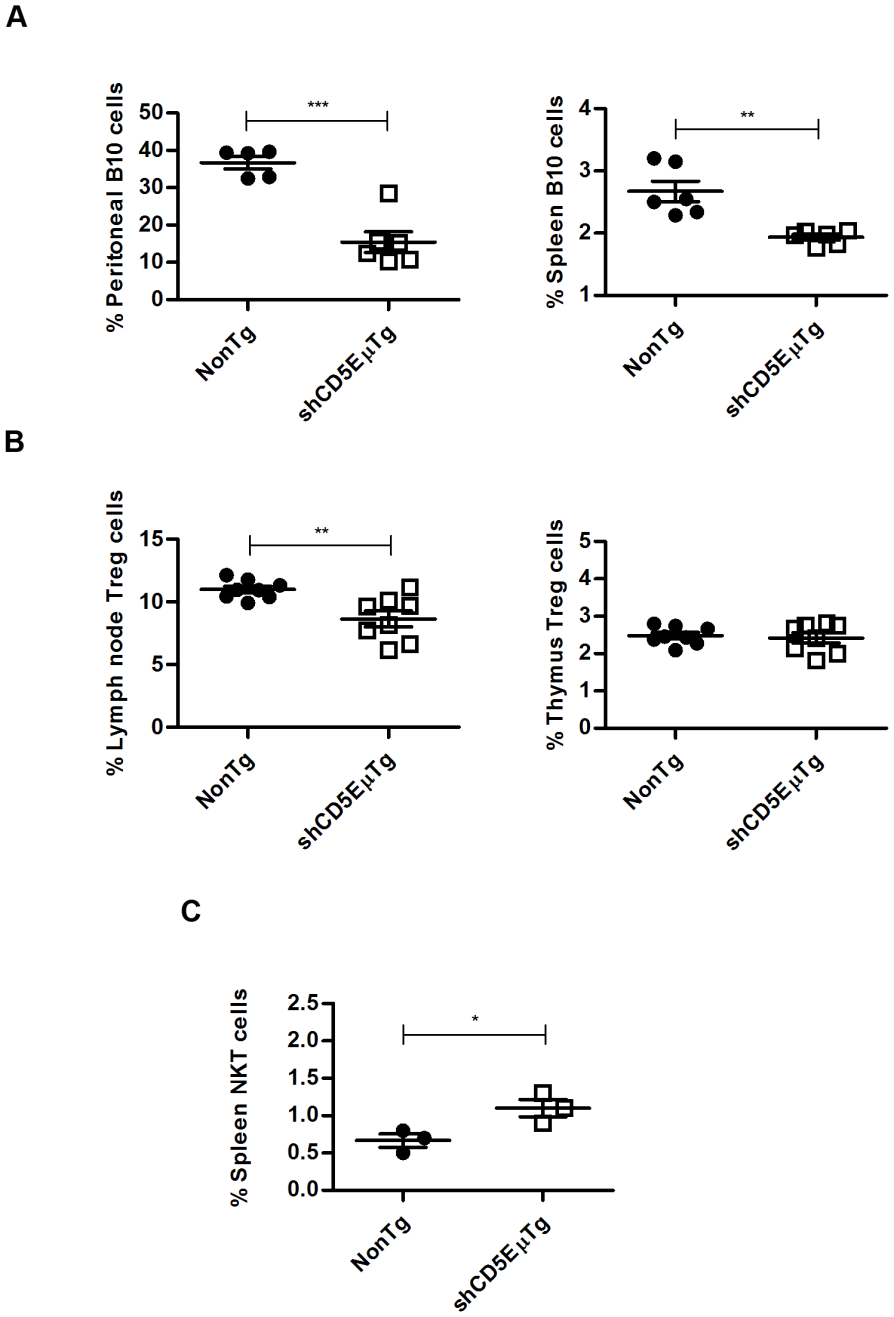
Flow cytometry analysis of T and B cells with regulatory phenotype in shCD5EμTg mice. A) Cells from peritoneal lavage and spleen samples from shCD5EμTg mice and non-transgenic littermates were stained for the presence of Breg (B10) cells (B220+CD1d+CD5+IL10+). *** p≤0.0006 ** p = 0.0015. B) Lymph node and thymus cells were stained for the presence of Treg cells (CD4+CD25+ Foxp3+) in shCD5EμTg mice and in non-transgenic littermates ** p = 0.0038. C) Spleen cells were stained to detect NKT cells (CD3+NK1.1+) in shCD5EμTg mice and non-transgenic littermates. * p = 0.0406.

Given that CD5+ B cells appeared to be reduced in shCD5EμTg mice, a subset of IL-10 secreting B cells with a regulatory/suppressor function, known as B10 cells (B220^+^ CD1d^hi^ CD5^+^ IL10^+^) [34], [35], was further analyzed. As shown in **Figure 4A** **and Figure S7A**, B10 cells were significantly decreased in both peritoneal lavage and spleen samples from shCD5EμTg mice compared with non-transgenic littermates, suggesting that intact CD5-mediated interactions are needed for the maintenance of this subpopulation.

It has recently been reported that CD5 expression regulates the generation and/or function of T regulatory cells (Tregs) [7], [36]. Since shCD5 might interfere with putative ligand binding of endogenous CD5 expressed on T lymphocytes, we investigated whether changes in Treg cell numbers could be detected in primary and secondary lymphoid organs of shCD5EμTg mice. As shown in **Figure 4B** **and Figure S7B**, no differences in the percentage or number of Treg (CD4+ CD25+ FoxP3+) cells were observed in the thymuses of shCD5EμTg mice. However, a significant reduction in the percentage of Tregs was observed in the lymph nodes of these mice compared with their non-transgenic littermates.

NKT cells (NK1.1+ CD3+) constitute another CD5+ regulatory cell subset. As shown in **Figure 4C** **and Figure S7C**, and contrary to what was observed for B and T cell regulatory subsets, NKT cells were found to be increased in spleens from shCD5EμTg mice compared with non-transgenic littermates. Overall, the data indicate that shCD5EμTg mice present alterations in different regulatory cell subpopulations, which could have important consequences for the immune response of these mice.

**Figure 5:**
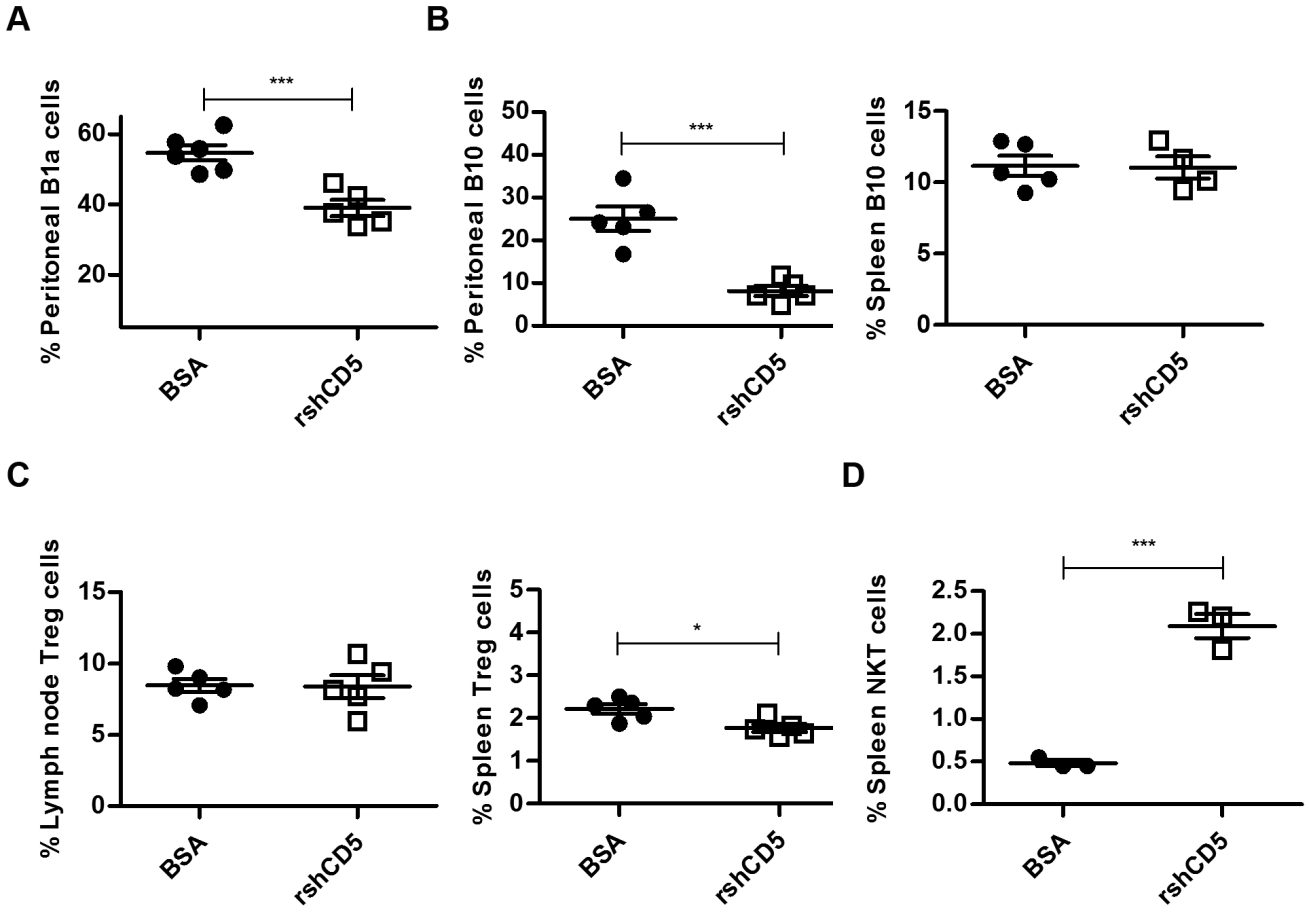
Flow cytometry analysis of T and B cells with regulatory phenotype in wild-type C57Bl/6 mice treated with exogenous rshCD5. A) B1a cells were analyzed as before in peritoneal lavage samples from wild-type C57Bl/6 mice treated with exogenous rshCD5 (25 µg every 48 hs for 14 days) or BSA as control. *** p = 0.0007. B) Cells from peritoneal lavage and spleen samples were stained for the presence of B10 cells in wild-type C57Bl/6 mice treated with exogenous rshCD5 (25 µg every 48 hs for 14 days) or BSA as control. C) Lymph node and spleen cells were stained for the presence of Treg cells in wild-type C57Bl/6 mice treated with exogenous rshCD5 (25 µg every 48 hs for 14 days) or BSA as control. * p = 0.0156. D) Spleen cells were stained to detect NKT cells in wild-type C57Bl/6 mice treated with rshCD5 (25 µg i.p., every 48 hs for 14 days) or BSA as control. *** p = 0.0004.

To rule out random events of transgenesis and confirm that the observed changes in regulatory cell subpopulations were caused by increased circulating levels of shCD5, similar cell analyses were performed on wild-type C57Bl/6 mice treated with repeated i.p. infusions of purified rshCD5 protein (1,25 mg/kg, every other day for two weeks). As shown in **Figures 5A and 5B**, B1a and B10 cells were significantly reduced in the peritoneum, but not in the spleen of rshCD5-treated mice compared with control mice. In addition, Treg cells were found to be reduced in the spleen but not in the lymph nodes of wild-type mice injected with rshCD5 (**Fig. 5C**). Finally, analysis of NK1.1+ cells, showed an increase in NKT cells (NK1.1+, CD3+) in the spleen of rshCD5-treated mice (**Fig. 5D**). These data indicate that sustained treatment of wild-type mice with exogenous rshCD5 can induce similar phenotypical changes to those observed in shCD5EμTg mice, unequivocally ascribing the effects of transgenesis to the presence of circulating shCD5.

### Antibody response to T-independent-1 antigens is enhanced in shCD5EμTg mice

**Figure 6:**
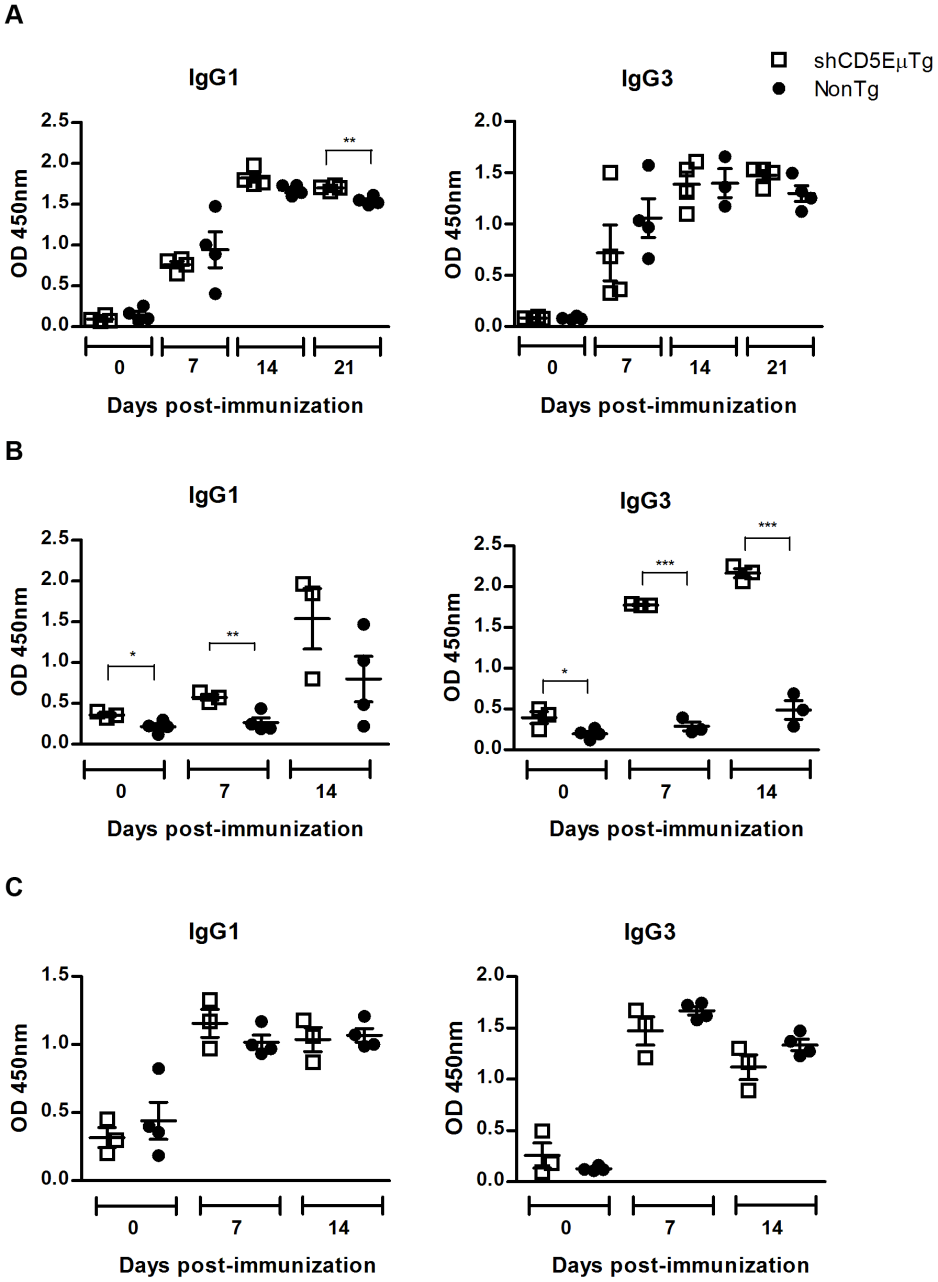
shCD5EμTg and non-transgenic mice response to immunization with T-dependent and T-independent (type 1 and type 2) antigens. Mice were immunized i.p. with 50 µg TNP5-KLH (A), TNP0.3-LPS (B) or TNP65-Ficoll (C) prepared in 400 µl of PBS in complete Freund's adjuvant at day 0 and in 400 µl vehicle at day 14, as examples of TD, TI type 1 and TI type 2 antigens, respectively. Sera from immunized mice were collected at days 0, 7 and 14 (for TI response) or 0, 7, 14 and 21 (for TD response) after the primary immunization and levels of TNP-specific antibodies determined by ELISA. Ig levels are expressed as OD 450 nm values. The bars represent the average value for each group. ** p<0.05 *** p<0.01.

To further evaluate the functional consequences of the lymphocyte subset alterations observed in shCD5EμTg mice, *in vivo* antibody responses to different T-dependent and –independent antigens were analyzed. Transgenic shCD5EμTg mice and non-transgenic littermates were immunized i.p. with the hapten trinitrophenol (TNP) conjugated to a T-dependent (TD) antigen (keyhole limpet hemocyanin; TNP_5_-KLH), a T-independent type 1 (TI-1) antigen (LPS; TNP_0,3_-LPS) and a T-independent type 2 (TI-2) antigen (Ficoll; TNP_65_-Ficoll), and serum levels of specific anti-TNP antibodies analyzed by ELISA. No significant differences were observed for anti-TNP antibody levels in shCD5EμTg mice immunized with TD antigen (TNP_5_-KLH), with the exception of IgG1 antibodies at day 21 post-immunization, which were significantly higher in transgenic mice (**Fig. 6A** and **Fig. S8A**). However, a trend towards higher levels of IgM antibodies was also observed in shCD5EμTg mice. Regarding the response to the TI-1 antigen (TNP_0,3_-LPS), higher levels of anti-TNP antibodies were also observed for shCD5 transgenic mice in all the Ig isotypes tested, at nearly all time points (**Fig. 6B** and **Fig. S8B**). By contrast, levels of antibodies in mice immunized with the TI-2 antigen (TNP_65_-Ficoll) appeared to be unchanged in shCD5EμTg mice at all time points, for all the different Ig isotypes analyzed (**Fig. 6C** and **Fig. S8C**). These results suggest that the B cell antibody response against TI-1 antigens – and, to a lesser extent, against TD antigens – is enhanced in shCD5EμTg mice compared with that of non-transgenic littermates.

### Experimentally induced autoimmune diseases are more severe in shCD5EμTg mice

**Figure 7:**
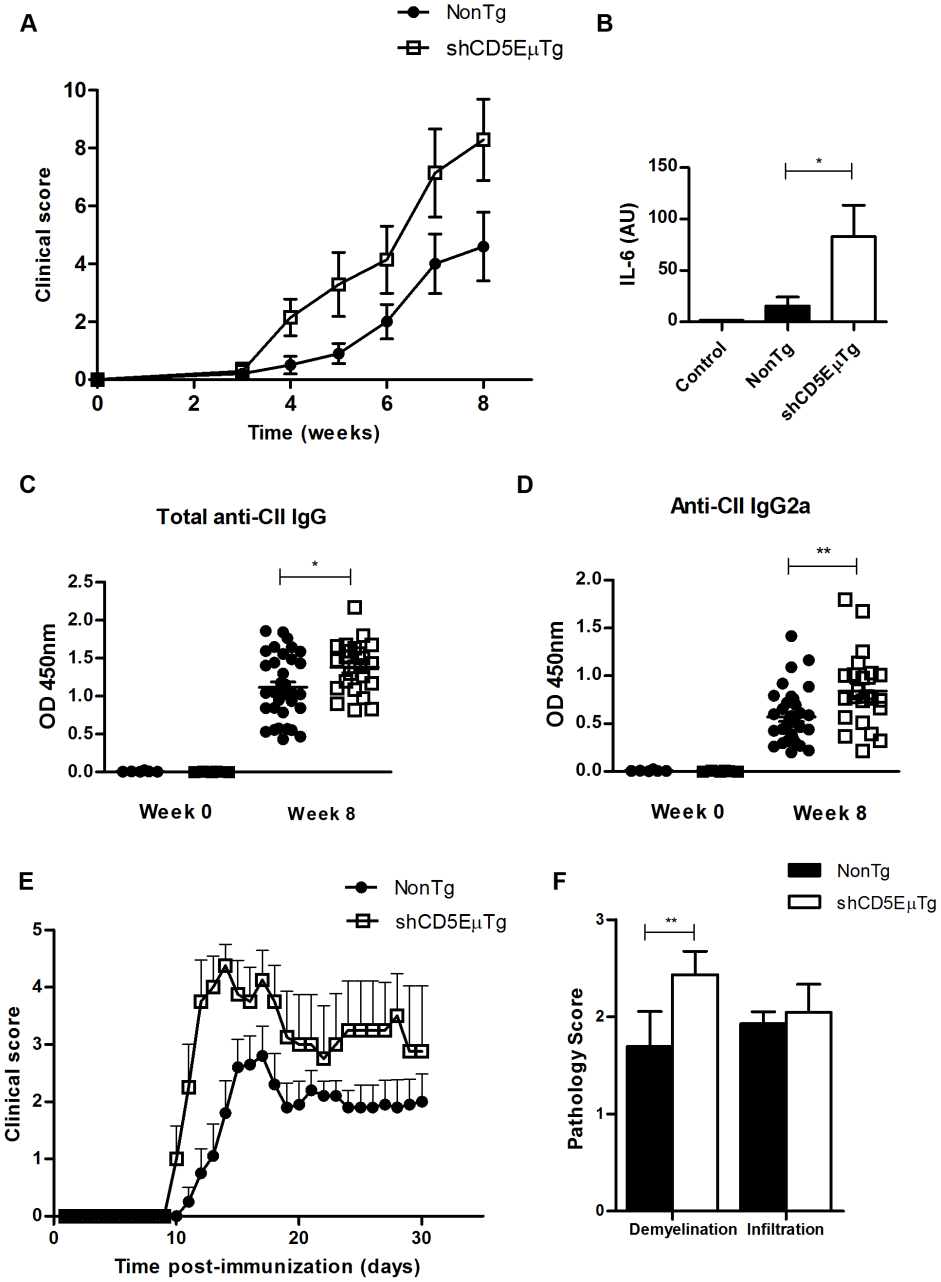
Exacerbated autoimmune disease in shCD5EμTg mice. A) Analysis of CIA induction in (DBA_B6)F1×shCD5EμTg mice and non-transgenic littermates. 7–8 week old mice were immunized s.c. near the base of the tail with 100 µl of antigen (2 mg/ml type II bovine collagen) emulsified in CFA plus Arthrogen-CIA adjuvant. Radiological signs were scored weekly for each mouse and results for each group averaged to determine overall clinical score. B) IL-6 levels were determined by real time RT-PCR in joint tissue, normalizing results to GAPDH expression levels. Results are expressed in arbitrary units (A.U.). * p = 0.05. C) and D) Antibodies against type II collagen were measured by ELISA in the sera of (DBA_B6)F1×shCD5EμTg transgenic mice and non-transgenic littermates. Antibody determination included total IgG (C) and IgG1 (D). Results are expressed as OD 450 nm. E) Analysis of EAE induction in shCD5EμTg and non-transgenic mice. 8–10 week old mice were administered s.c. with 200 µl of antigen (50 µg MOG peptide) in CFA. At the time of immunization and 48 hs later, 200 ng of Pertussis toxin were also injected i.p. Clinical score was determined daily and it represents the average value for each mice, where 0 = no disease; 0.5 = partially motionless tail; 1 = motionless tail; 2 = ataxia; 3 = loss of mobility in one limb; 4 = loss of mobility in back limbs; 5 = moribund. Graphs show the comparison between clinical scores of shCD5EμTg (n = 5) and non-transgenic mice (n = 10). p = 0.05 (Two-way ANOVA test). F) Demyelination and infiltration scores, as detected by solochrome cyanin and hematoxylin and eosin staining, respectively. ** p = 0.02.

To gain insight into the *in vivo* immune response to auto-antigens in shCD5EμTg mice, two different models of experimentally induced autoimmune disease were evaluated. First, collagen-induced arthritis (CIA) in (B6×DBA/1) F1 mice, a well-established model of T-cell mediated autoimmune disease resembling rheumatoid arthritis, was used. As shown in **Figure 7A** and **Figure S9A**, increased severity of arthritis symptoms (oedema, joint narrowing, marginal erosion, osteopenia) was observed in shCD5EμTg mice compared with non-transgenic littermates. Moreover, IL-6 levels were significantly increased in joint tissue from shCD5EμTg mice compared with that of non-transgenic mice, while a trend towards increased levels of other pro-inflammatory cytokines (IFN-γ, IL-1β, IL-17 and TNF-α) was observed (**Fig. 7B** and **Fig. S9B**). Total anti-collagen II antibody levels were also increased in the sera of shCD5EμTg mice, mostly of the IgG2 subtype, (**Fig. 7C and 7D** and **Fig. S9C**), characteristic of an enhanced Th1 response.

To further validate the results obtained with the CIA model, the MOG-induced model of experimental autoimmune encephalomyelitis (EAE), a well-established model of T-cell mediated autoimmune disease resembling multiple sclerosis, was evaluated. As shown in **Figure 7E**, the clinical score of the disease was significantly increased in shCD5EμTg mice compared with non-transgenic littermates. Moreover, demyelination was also increased in shCD5EμTg mice (**Fig. 7F**). Altogether, the data show that shCD5EμTg mice display more severe forms of experimentally induced autoimmune disease in models developed against different auto-antigens.

### Tumor growth is slowed in shCD5EμTg mice

**Figure 8:**
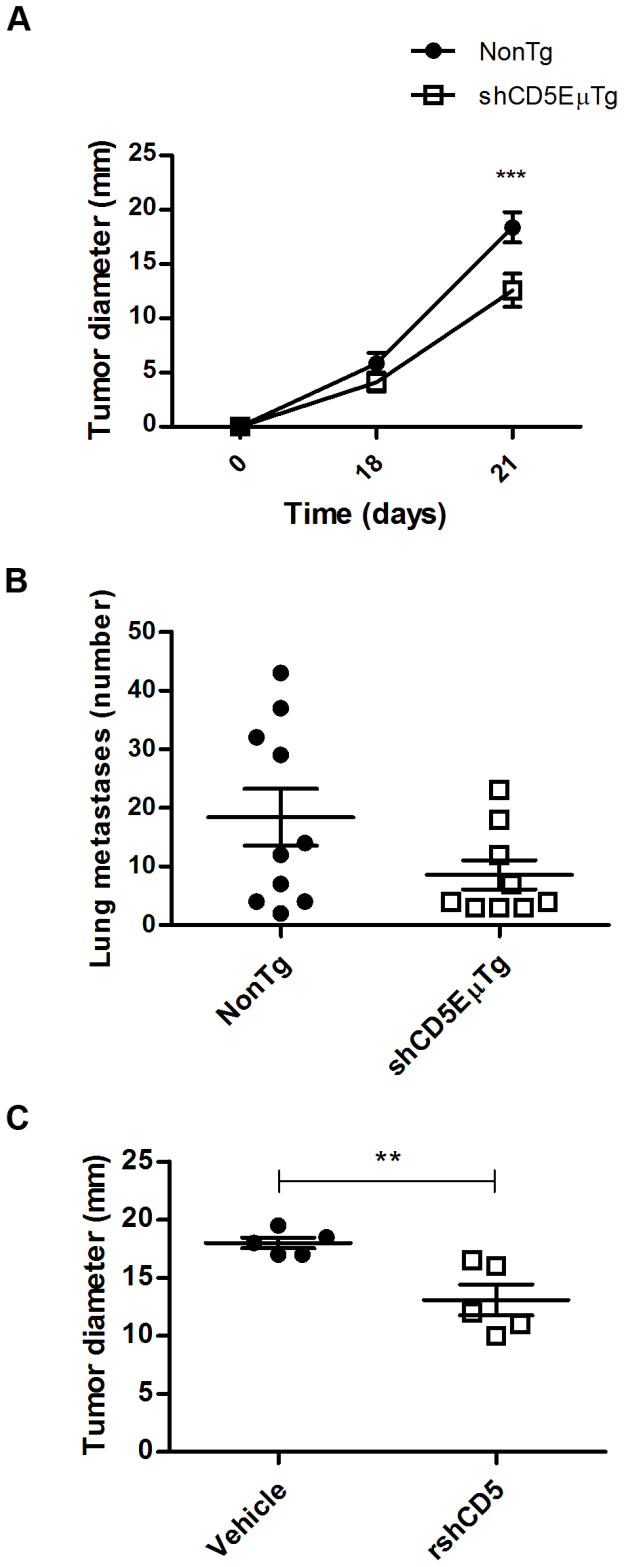
Delayed tumour growth in shCD5EμTg mice. A) shCD5EμTg mice (n = 16) and non-transgenic littermates (n = 15) were injected s.c. with 5×10^4^ B16 melanoma cells. Tumour growth was recorded over time by measuring with a Vernier calliper. *** p = 0.0177. B) Experimental metastasis assay. Mice were injected i.v. with 1×10^5^ B16 melanoma cells. Fifteen days later, mice were sacrificed, their lungs removed and the metastases on the surface counted. p = 0.0994. C) Non-transgenic C57Bl/6 mice (n = 5 for each group) were injected with B16 cells as before. Chemotherapy (vincristine 0.5 mg/kg, Doxorubicin 3.3 mg/kg) was administered i.p. at day 3 after tumor cell injection. Additionally, rshCD5 (25 µg) or vehicle was injected i.p. every 48 hs for the duration of the experiment (20 days). ** p = 0.0083.

Since autoimmune and anti-tumoral immune responses are both directed mainly against self-antigens, the *in vivo* response of shCD5EμTg mice to autologous tumor antigens was evaluated. A melanoma B16 cell model (syngeneic with C57Bl/6 mice) was chosen, and tumor growth was recorded over time. As shown in **Figure 8A**, B16 tumors grew significantly more slowly in shCD5EμTg mice compared with their non-transgenic littermates. Taking these data into account, a potentially improved response of shCD5EμTg mice to i.v. injected B16 cells was also evaluated. Experimental metastasis assays revealed a decrease in the number of lung metastases observed in shCD5EμTg compared with non-transgenic littermates (**Fig. 8B**), which was close to significance (p = 0.09).

To evaluate whether administration of exogenous rshCD5 to non-transgenic mice could also delay tumor growth, wild-type C57Bl/6 mice s.c. implanted with B16 cells were treated with vehicle (PBS+glycerol) or rshCD5 (1,25 mg/kg) administered every 48 h from the day of tumor cell injection. A single dose of chemotherapy (vincristine 0.5 mg/kg, doxorubicin 3.3 mg/kg) was also administered at day 3 after tumor cell injection for both groups. As shown in **Figure 8C**, tumor progression was slowed in mice treated with chemotherapy plus rshCD5, compared with mice treated with chemotherapy plus vehicle. Altogether, these results suggest that increased circulating levels of shCD5 delay progression of melanoma cells in mouse tumor models.

## Discussion

Important immunomodulatory properties have been ascribed to CD5 during lymphocyte development and activation [37], which make this receptor a potential target for immunotherapeutic approaches. Although no CD5 deficiency has been reported in humans, studies in CD5−/− mice have provided valuable information in this regard, showing CD5 to act as a negative modulator of antigen-driven activation of T cells (and certain B cells) [15], [16]. Considering that genetic deletion or silencing of CD5 in humans would be fraught with both technical and ethical problems, induction of sustained circulating levels of a soluble form of CD5 was devised as an alternative strategy of hindering this receptor in a functional and reversible way. In order to avoid repeated infusions of expensive purified protein and also to maintain stable levels of soluble CD5, a new shCD5-expressing transgenic mouse line (shCD5EμTg) was generated, which may serve as a test-bed for future applications in the clinic.

The shCD5EμTg mice herein reported display modest, albeit readily detectable and sustainable serum levels of shCD5 from early stages of development, making the mice tolerant to the human protein. To develop this transgenic mouse line, advantage was taken of the inter-species conservation of the receptor-ligand interactions existing between mouse and human CD5, something which had already been reported for the closely related receptor CD6 [33], [38]. Indeed, shCD5 is able to interact with the as yet undefined CD5 ligand/s, thus likely acting as a decoy receptor capable of impairing the functional consequences of receptor-ligand interactions mediated by membrane-bound CD5. Thus, shCD5EμTg mice would have a lower threshold for antigen-specific receptor-mediated activation and subsequently, an enhanced immune response. This is confirmed in this work, where shCD5EμTg mice are shown to have an increased response against different types of antigens.

Transgenic shCD5EμTg mice were shown to display an enhanced *in vivo* antibody response to certain soluble antigens, particularly TD and TI-1 antigens. Indeed, induction of two different experimental autoimmune diseases (CIA and EAE) resulted in a more severe outcome for shCD5EμTg mice compared with non-transgenic littermates. The increase in disease severity was measured not only by clinical score, but also through determination of pro-inflammatory cytokine levels and autoantibody responses. While no CIA studies have been carried out in CD5−/− mice, it has been recently shown that CD5−/− and CD5ΔCKIIBD mice (mice expressing a truncated and functionally defective form of CD5) immunized with high doses of MOG show more severe forms of EAE than wild-type mice [39]. Thus, physical deletion and functional knock-down of CD5 both appear to lead to a similar outcome with regard to autoimmune responses.

Both autoimmune and anti-tumor responses are directed mainly towards autologous antigens. Indeed, syngeneic B16 melanoma tumors developed in shCD5EμTg and in non-transgenic mice, but grew at a slower rate in shCD5EμTg mice, which also showed a decreased number of lung metastases following i.v. injection of tumor cells. These results are also in agreement with previous studies carried out in CD5−/− mice, in which B16F10 tumors grew more slowly compared to those in wild-type mice [40].

Phenotypical analysis of shCD5EμTg mice did not reveal any gross alterations in major lymphocyte subpopulations. However, careful subpopulation analysis showed a decrease in T and B cell subsets with regulatory function, as well as an increase in NKT cells. It has been reported that generation of thymus-derived T regulatory cells (nTregs) – which express high levels of membrane-bound CD5 - appears to be regulated by CD5 expression [7], [36]. In shCD5EμTg mice, Treg cells (CD4+CD25+FoxP3+) were decreased in the periphery but not in the thymus. This could be explained by the fact that shCD5 expression is under the control of the Eμ enhancer and is thus expressed preferentially by B cells. Local expression in the thymus would thus be very low and might not be enough to alter the development or maintenance of nTreg cells, suggesting that the observed decrease in Treg cells is at the expense of inducible Treg (iTregs) cells. Another possibility is that cross-linking of membrane-bound CD5 is not necessary for T cell development in the thymus, as it has been reported that the cytoplasmic region alone might be sufficient for intrathymic selection [41]; this is consistent with the authors mentioning the lack of CD5L expression in the thymus. However, the authors also point out that CD5-CD5L interactions would indeed be important in the periphery, which is in agreement with our results. Thus, although the extracellular region of CD5 might be dispensable for some of its functions, it is still necessary for modulation of antigen-specific receptor signalling, at least in the periphery.

B10 cells were also decreased in shCD5EμTg mice. The same subpopulation is also decreased in CD5−/− and in CD5ΔCKIIBD mice (data not shown), supporting an important role for intact CD5 function in the maintenance of this subpopulation [42]. Indeed, it has been reported that CD5 expression is required for IL-10 production by Bregs, acting as a survival factor [42]. In a similar way, intact CD5 function might be required for other CD5-expressing cell types, such as NKT cells. It is also possible that NKT cell number and/or activity could be influenced indirectly by regulatory T and/or B cells, as has been speculated previously [43].

Importantly, the observed changes in regulatory lymphocyte subpopulations were not due to random transgene insertion, since the phenotype could be reproduced in wild-type C57Bl/6 mice by repeated administration of exogenous rshCD5 protein. These results indicate that there is a causal relationship between the presence of increased circulating levels of shCD5 and changes in cell subpopulation dynamics, particularly in those cell types with high membrane-bound CD5 expression.

The results obtained in functional assays (autoimmunity and tumor models) tie in well with the decrease in lymphocyte subpopulations with regulatory/suppressor function observed in shCD5EμTg mice. Regulatory cells have been shown to suppress activation of the immune system, and a decrease in these cells would then be expected to enhance the immune response to self and non-self antigens, particularly when coupled with an increase in NKT cells. The latter cells are able to generate large quantities of cytokines that can quickly modulate and polarize the immune response. However, a decrease in Treg cells by itself has been reported to slow down tumor growth [44].

Although the main intention of these experiments was to characterize the immune response in shCD5EμTg mice, these mice were also developed to test potential future clinical applications of shCD5. As such, our results suggest a beneficial effect of shCD5 in the treatment of tumors, through an enhancement in the *in vivo* anti-tumor response. Cancer treatment, however, is usually a combination of different drugs and/or therapies; if shCD5 were ever to be used in the treatment of tumors, it would probably be as an adjuvant to an established therapy such as chemotherapy. Our results show that exogenous repeated administration of rshCD5 in combination with chemotherapy improved the performance of chemotherapy alone in mice, suggesting that this protein might be used as an adjuvant of standard chemotherapy.

In conclusion, impairment of CD5/CD5L interactions by shCD5 results in an enhancement of the immune response against autologous antigens. This enhancement could be due to the observed changes in lymphocyte subpopulations with regulatory function; another possibility is that, considering that CD5 is a negative regulator of T and B cell responses, impairment of its function could also result in enhanced activation of effector T and B cells. Although it is not clear which mechanism is prevalent, it is likely that both effects contribute to the overall phenotype in a combined manner. The results therefore suggest that shCD5 is an immunomodulatory target that could be used in the treatment of diseases which would benefit from an enhancement of a normal or depressed immune response, such as cancer.

## Supporting Information

Figure S1 **Interespecies cross-reactivity of receptor-ligand interactions mediated by CD5 and CD6.** Mouse (top panels) and human (bottom panels) PBMCs (1.5×10^5^) were cultured for 72 hs in the presence (bold line) or absence (fine line, gray filling) of PMA 100 ng/ml and Ionomycin 1 µg/ml. Cells were then incubated with 1 µg biotin-labeled shCD5 (left panels) or shCD6 (right panels) for 1 hr, and developed with FITC-conjugated streptavidin for further analysis by flow cytometry. Results shown correspond to the gated lymphocyte subpopulation.
(TIF)

Figure S2 **Identification of shCD5EμTg mice by PCR.** Following PCR amplification of shCD5 from tail DNA, a PCR product of the expected size (450 bp) was detected in transgenic mice. A fragment of 150 bp corresponding to the LIEX gene was also amplified as an internal control for the PCR.
(TIF)

Figure S3 **Analysis of major T lymphocyte subpopulations and development in shCD5EμTg mice.** Lymph node (A) and spleen (B) cells were stained with anti-CD4 and anti-CD8 specific antibodies to identify CD4−, CD8−, or CD4−CD8− (mostly B cells). C) To analyze the effect of shCD5 expression on T cell development, thymocytes were stained with anti-CD4 and anti-CD8 antibodies to identify DN, DP, CD4+SP and CD8+SP cells. D) To analyze the effect of shCD5 expression on early T cell development, thymocytes were stained with anti-CD44 and anti-CD25 antibodies to distinguish between DN1 (CD44+CD25−), DN2 (CD44+CD25+), DN3 (CD44−CD25+) and DN4 (CD44−CD25−) cells.
(TIF)

Figure S4 **Flow cytometry analysis of B lymphocyte subpopulations in shCD5EμTg mice.** To analyze peripheral B cell subsets from spleen and peritoneum, we used a gating strategy adapted from Cariappa *et al* [45]. Transitional 3 (T3) and follicular I (FOLI) cells can be distinguished in the IgM^low^IgD^high^ gated subpopulation using the CD21/35 and CD93/AA4 markers. Finally, the IgM^high^IgD^high^ gated subpopulation allows for the detection of follicular II (FOLII) cells and marginal zone precursors (MZP) based on the surface expression of AA4 and CD21.
(TIF)

Figure S5 **Gating for flow cytometry analysis of B lymphocyte subpopulations in shCD5EμTg mice.** A) Representative dot plots showing the gating strategy for B cell supopulation analysis. First, cells were gated for IgM and IgD; subsequently, T1, T2 and MZ cells were detected in the IgM^high^IgD^low^ gate by staining with CD93/AA4 and CD93. Similarly, gated on IgM^low^IgD^high^, FOL I and T3 cells could be distinguished according to CD21 and CD93/AA4 staining. Finally, gating on IgM^high^IgD^high^ allowed for detection of FOL II and MZP based on CD93/AA4 and CD21/35 staining. B) Representative dot plots showing the gating strategy for B1 and B2 subpopulation analysis. Gated on IgM^high^IgD^low^, B1a and B1b cells can be distinguished based on their CD5 expression, while B2 cells are IgM^high^IgD^high^.
(TIF)

Figure S6 **Analysis of B lymphocyte development in shCD5EμTg mice.** To analyze the effect of shCD5 in B lymphocyte development, bone marrow cells were stained with a series of antibodies (CD43, B220, CD24 and BP-1) to identify the different B cell subsets at various stages of maturation. From the gated CD43+B220+ subpopulation, pre-pro-B cells (CD24^low^BP-1−), early pro-B cells (CD24+BP-1−), late pro-B cells (CD24+BP-1+) and pre-B cells (CD24^high^BP-1+) could be identified based on the expression of CD24 and BP-1.
(TIF)

Figure S7 **Gating for flow cytometry analysis of regulatory lymphocyte subpopulations in shCD5EμTg mice.** A) Representative dot plots showing the gating strategy for B10 cell analysis. Cells gated on CD1d+CD5+ were analyzed for B220 and IL-10 staining; B220+IL-10+ cells were considered as B10 cells. B) Representative dot plots showing the gating strategy for Treg cell analysis. Gated on CD4+, CD25+FoxP3+ cells were considered as Treg cells. C) Representative dot plots showing the gating strategy for NKT cell analysis. CD3+NK1.1+ cells were considered as NKT cells.
(TIF)

Figure S8 **shCD5EμTg and non-transgenic mice IgM response to immunization with T-dependent and T-independent (type 1 and type 2) antigens.** Mice were immunized i.p. with 50 µg TNP5-KLH (A), TNP0.3-LPS (B) or TNP65-Ficoll (C) in 200 µl PBS, as examples of TD, TI type 1 and TI type 2 antigens, respectively. Sera from immunized mice were collected at days 0, 7 and 14 (for TI response) or 0, 7, 14 and 21 (for TD response) after the primary immunization and levels of TNP-specific antibodies determined by ELISA. IgM levels are expressed as OD 450 nm values. The bars represent the average value for each group. ** p<0,05.
(TIF)

Figure S9 **Exacerbated autoimmune disease in shCD5EμTg mice.** A) Radiological signs (edema, joint narrowing, marginal erosions and osteopenia) were scored at week 8 for each mouse as described in the Materials and Methods section. B) Cytokine mRNA levels were determined by real time RT-PCR in joint tissue, normalizing results to GAPDH expression levels. Results are expressed in arbitrary units (A.U.). C) IgG1 antibodies against type II collagen were measured by ELISA in the sera of (DBA_B6)F1×shCD5EμTg transgenic mice and non-transgenic littermates at weeks 0 and 8. Results are expressed as OD 450 nm.
(TIF)
