# Supplementary figures and images for: Transgenic Expression of Soluble Human CD5 Enhances Experimentally-Induced Autoimmune and Anti-Tumoral Immune Responses
Source: PLoS One. 2014 Jan 15;9(1):e84895. doi: 10.1371/journal.pone.0084895 (PMC3893160; doi:10.1371/journal.pone.0084895)

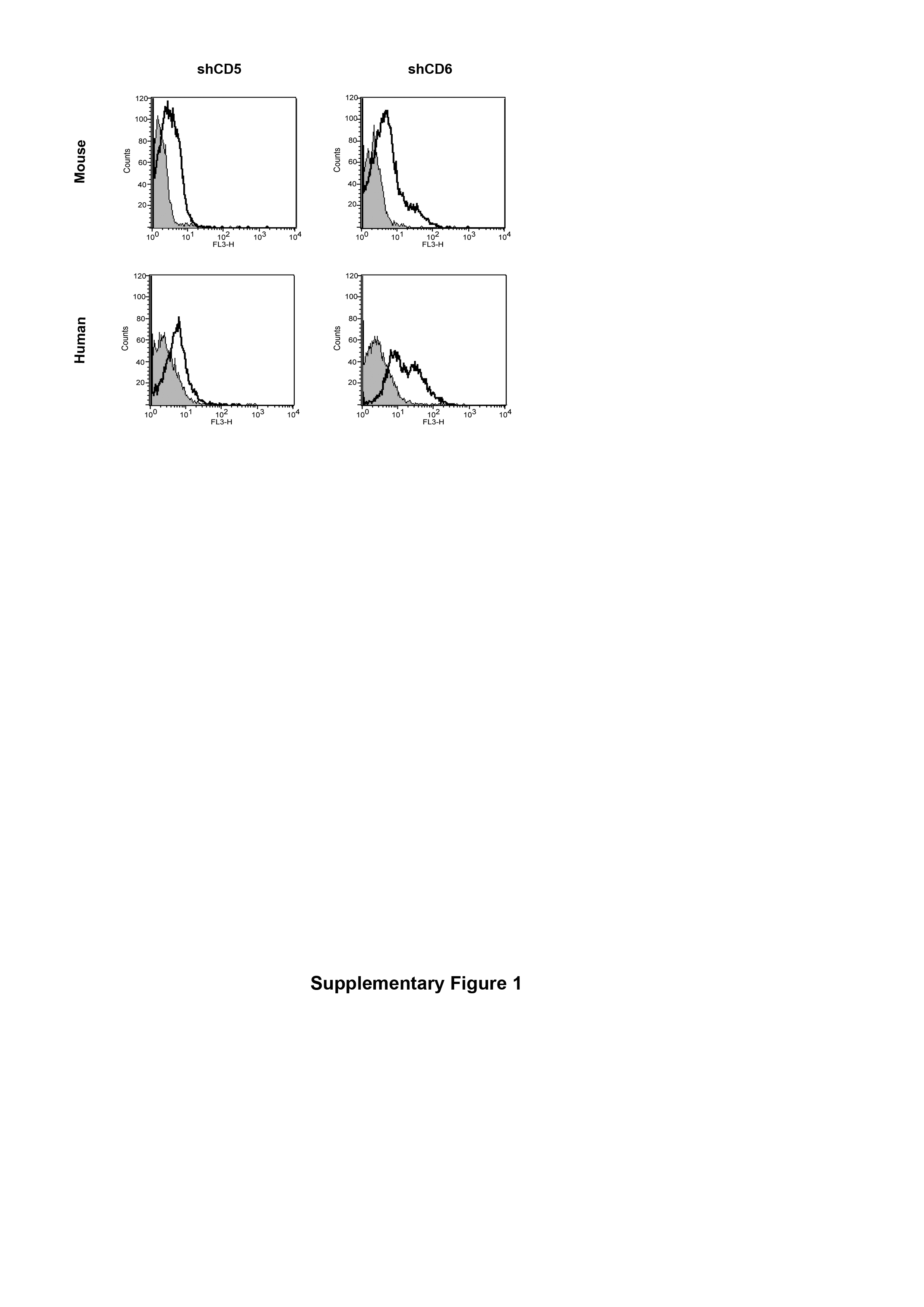

Supplement: Figure S1 — Interespecies cross-reactivity of receptor-ligand interactions mediated by CD5 and CD6. Mouse (top panels) and human (bottom panels) PBMCs (1.5×10^5^) were cultured for 72 hs in the presence (bold line) or absence (fine line, gray filling) of PMA 100 ng/ml and Ionomycin 1 µg/ml. Cells were then incubated with 1 µg biotin-labeled shCD5 (left panels) or shCD6 (right panels) for 1 hr, and developed with FITC-conjugated streptavidin for further analysis by flow cytometry. Results shown correspond to the gated lymphocyte subpopulation. (TIF) [file pone.0084895.s001.tif]

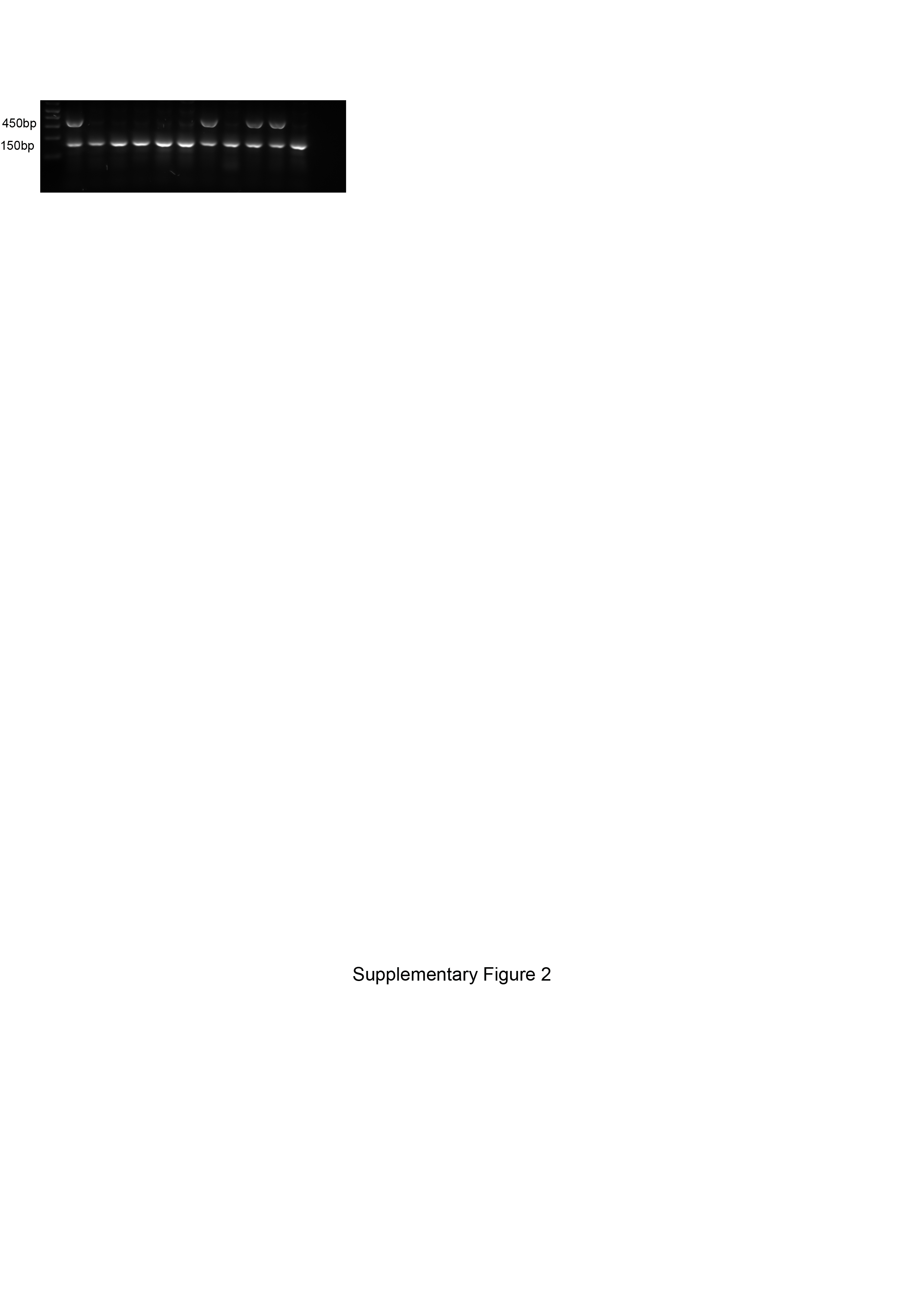

Supplement: Figure S2 — Identification of shCD5EμTg mice by PCR. Following PCR amplification of shCD5 from tail DNA, a PCR product of the expected size (450 bp) was detected in transgenic mice. A fragment of 150 bp corresponding to the LIEX gene was also amplified as an internal control for the PCR. (TIF) [file pone.0084895.s002.tif]

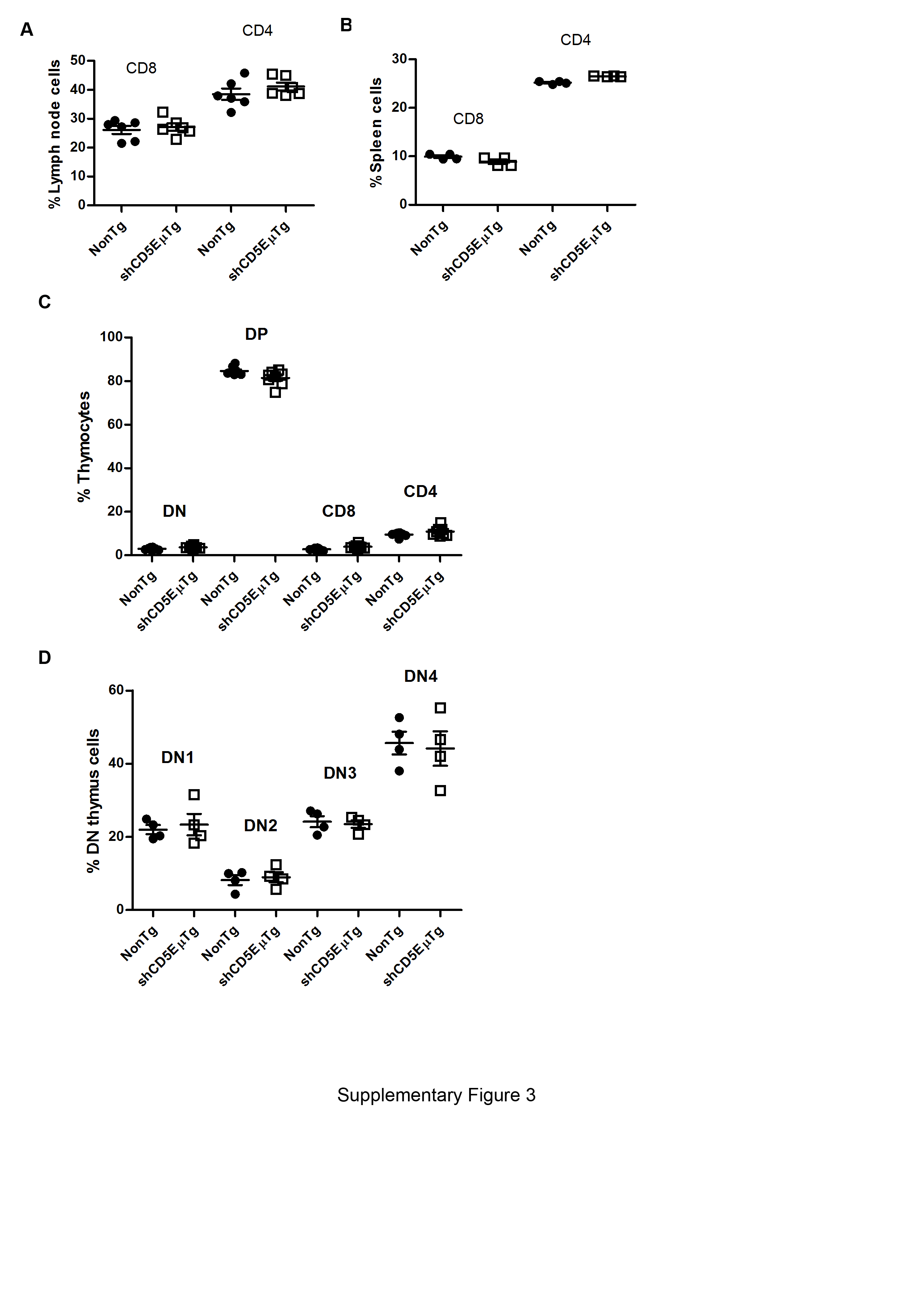

Supplement: Figure S3 — Analysis of major T lymphocyte subpopulations and development in shCD5EμTg mice. Lymph node (A) and spleen (B) cells were stained with anti-CD4 and anti-CD8 specific antibodies to identify CD4−, CD8−, or CD4−CD8− (mostly B cells). C) To analyze the effect of shCD5 expression on T cell development, thymocytes were stained with anti-CD4 and anti-CD8 antibodies to identify DN, DP, CD4+SP and CD8+SP cells. D) To analyze the effect of shCD5 expression on early T cell development, thymocytes were stained with anti-CD44 and anti-CD25 antibodies to distinguish between DN1 (CD44+CD25−), DN2 (CD44+CD25+), DN3 (CD44−CD25+) and DN4 (CD44−CD25−) cells. (TIF) [file pone.0084895.s003.tif]

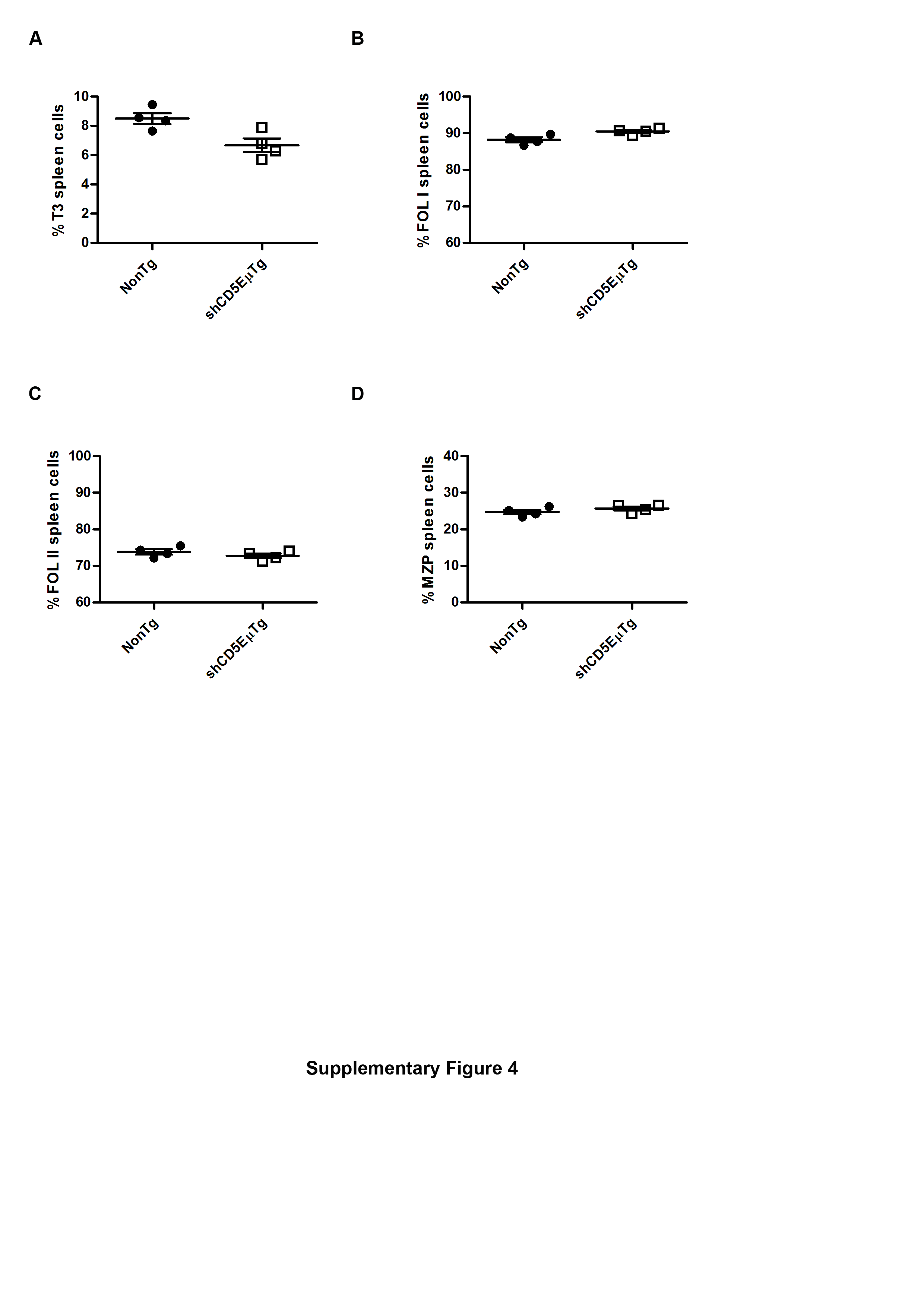

Supplement: Figure S4 — Flow cytometry analysis of B lymphocyte subpopulations in shCD5EμTg mice. To analyze peripheral B cell subsets from spleen and peritoneum, we used a gating strategy adapted from Cariappa et al [45]. Transitional 3 (T3) and follicular I (FOLI) cells can be distinguished in the IgMlowIgDhigh gated subpopulation using the CD21/35 and CD93/AA4 markers. Finally, the IgMhighIgDhigh gated subpopulation allows for the detection of follicular II (FOLII) cells and marginal zone precursors (MZP) based on the surface expression of AA4 and CD21. (TIF) [file pone.0084895.s004.tif]

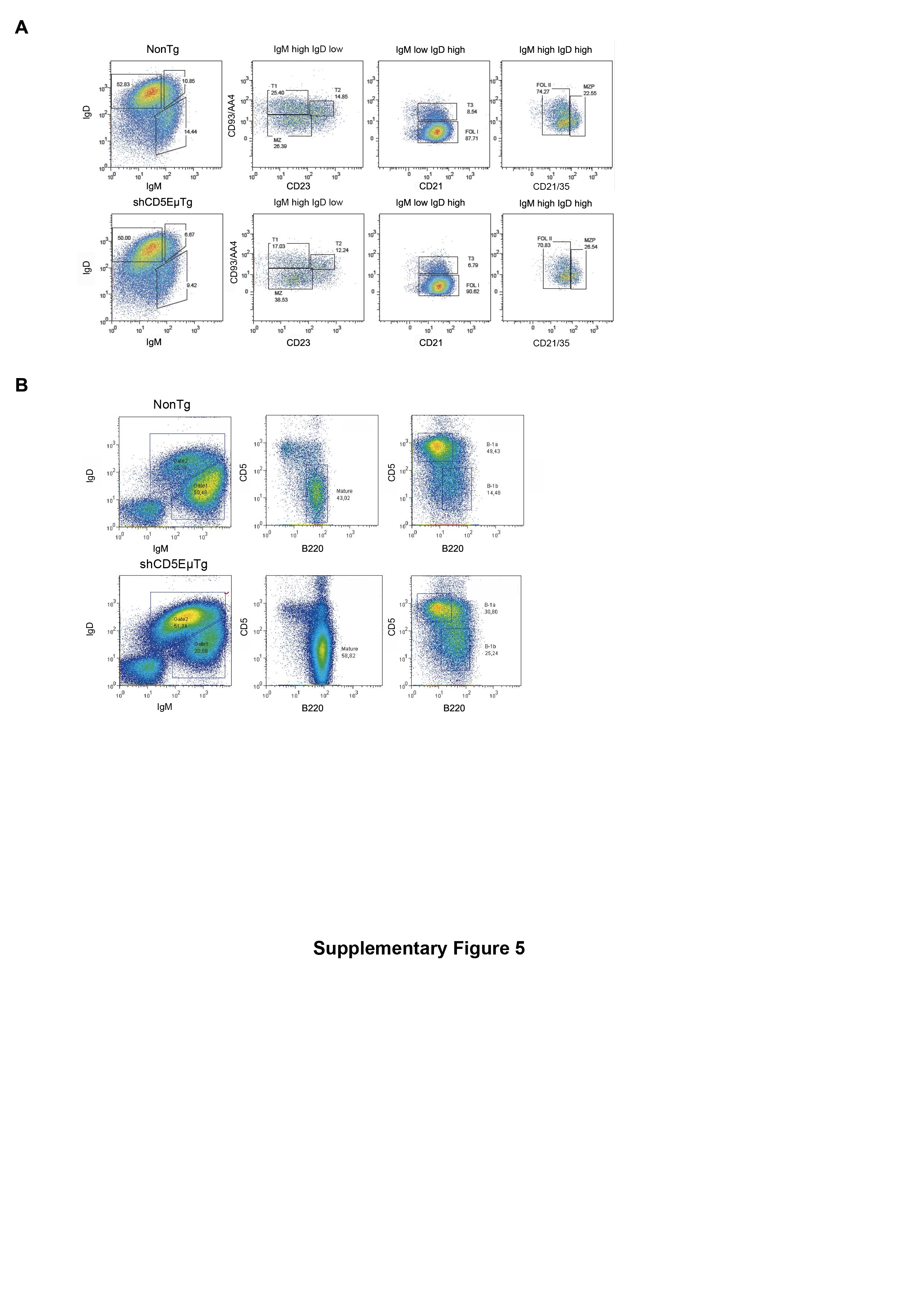

Supplement: Figure S5 — Gating for flow cytometry analysis of B lymphocyte subpopulations in shCD5EμTg mice. A) Representative dot plots showing the gating strategy for B cell supopulation analysis. First, cells were gated for IgM and IgD; subsequently, T1, T2 and MZ cells were detected in the IgMhighIgDlow gate by staining with CD93/AA4 and CD93. Similarly, gated on IgMlowIgDhigh, FOL I and T3 cells could be distinguished according to CD21 and CD93/AA4 staining. Finally, gating on IgMhighIgDhigh allowed for detection of FOL II and MZP based on CD93/AA4 and CD21/35 staining. B) Representative dot plots showing the gating strategy for B1 and B2 subpopulation analysis. Gated on IgMhighIgDlow, B1a and B1b cells can be distinguished based on their CD5 expression, while B2 cells are IgMhighIgDhigh. (TIF) [file pone.0084895.s005.tif]

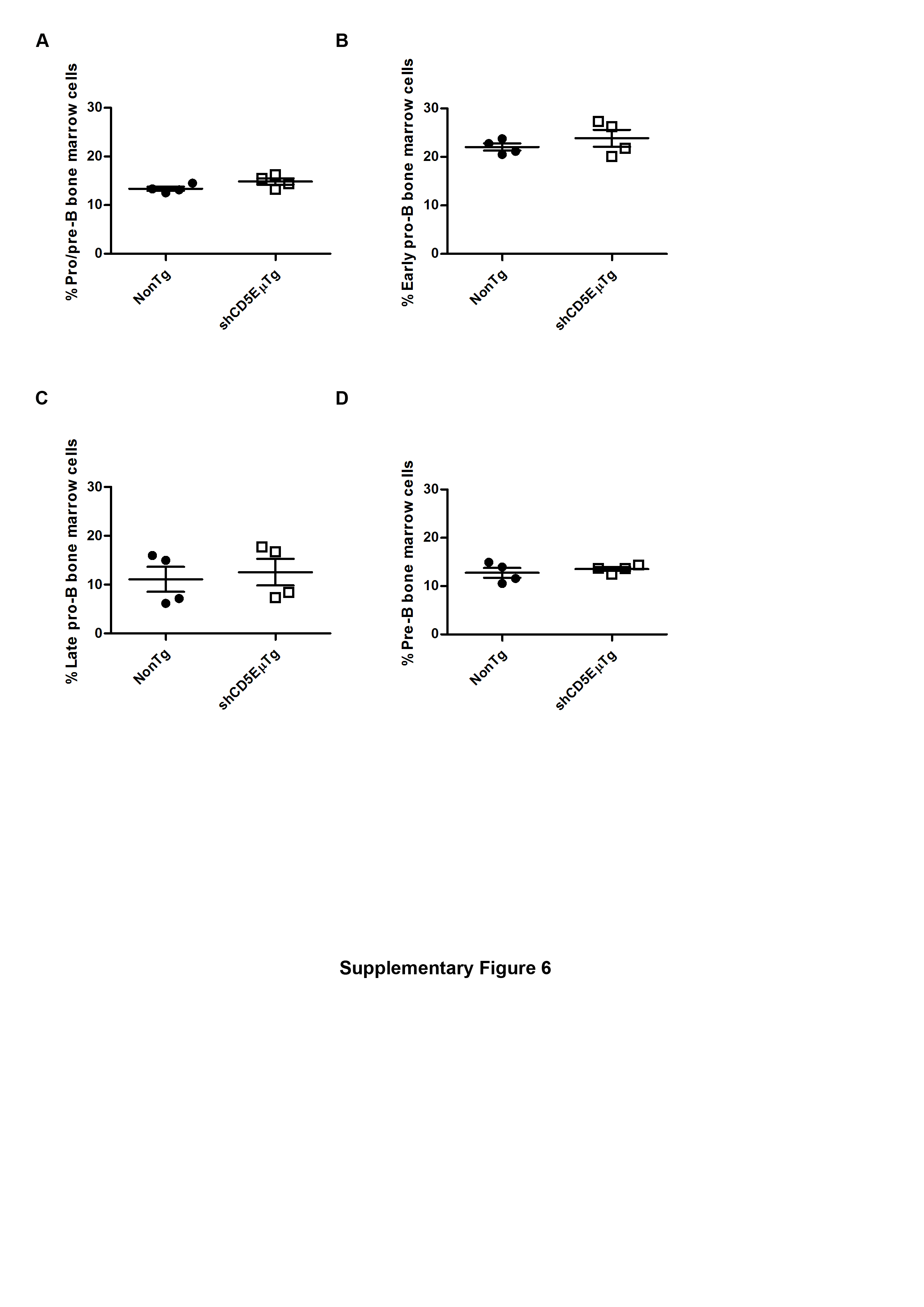

Supplement: Figure S6 — Analysis of B lymphocyte development in shCD5EμTg mice. To analyze the effect of shCD5 in B lymphocyte development, bone marrow cells were stained with a series of antibodies (CD43, B220, CD24 and BP-1) to identify the different B cell subsets at various stages of maturation. From the gated CD43+B220+ subpopulation, pre-pro-B cells (CD24lowBP-1−), early pro-B cells (CD24+BP-1−), late pro-B cells (CD24+BP-1+) and pre-B cells (CD24highBP-1+) could be identified based on the expression of CD24 and BP-1. (TIF) [file pone.0084895.s006.tif]

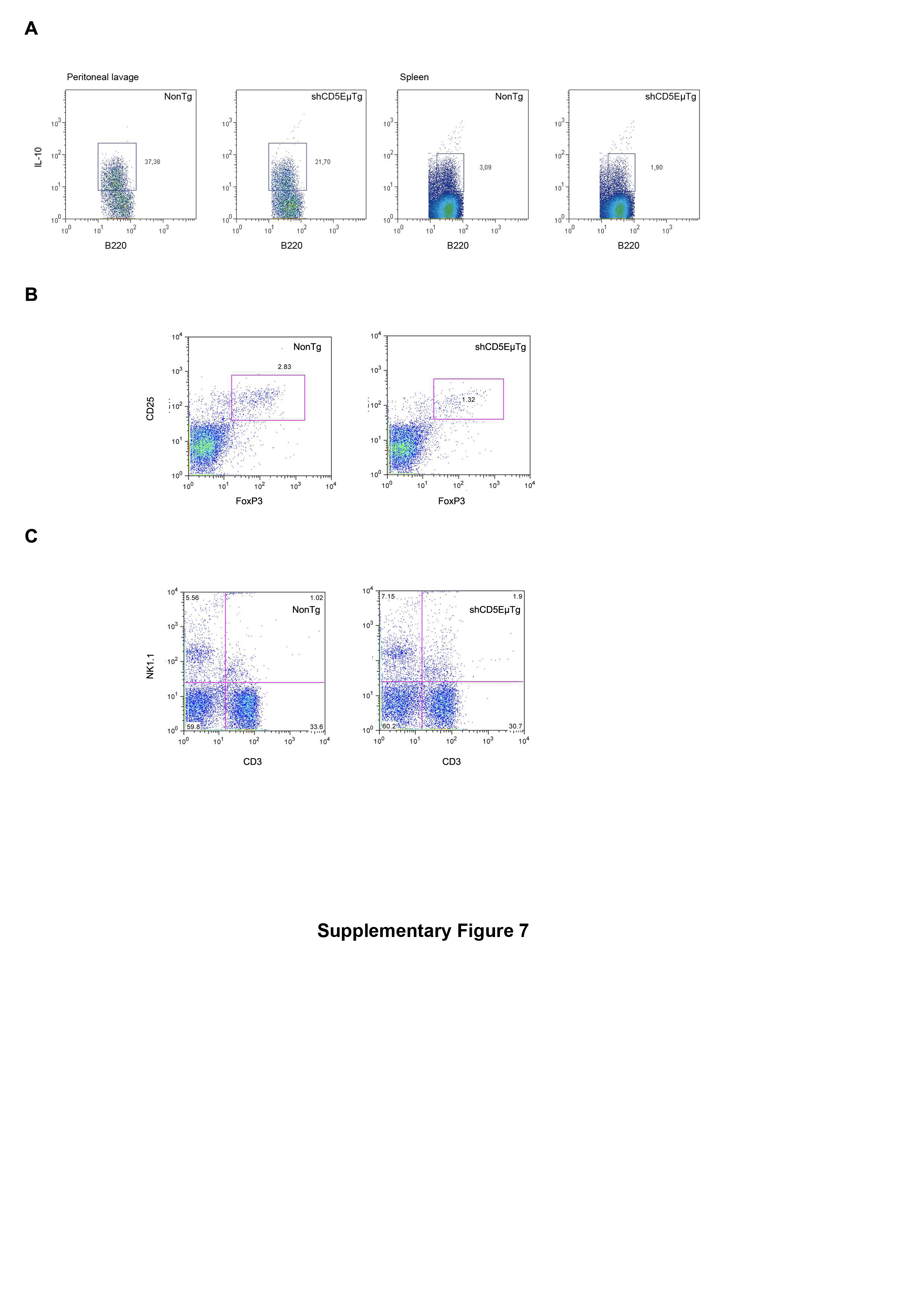

Supplement: Figure S7 — Gating for flow cytometry analysis of regulatory lymphocyte subpopulations in shCD5EμTg mice. A) Representative dot plots showing the gating strategy for B10 cell analysis. Cells gated on CD1d+CD5+ were analyzed for B220 and IL-10 staining; B220+IL-10+ cells were considered as B10 cells. B) Representative dot plots showing the gating strategy for Treg cell analysis. Gated on CD4+, CD25+FoxP3+ cells were considered as Treg cells. C) Representative dot plots showing the gating strategy for NKT cell analysis. CD3+NK1.1+ cells were considered as NKT cells. (TIF) [file pone.0084895.s007.tif]

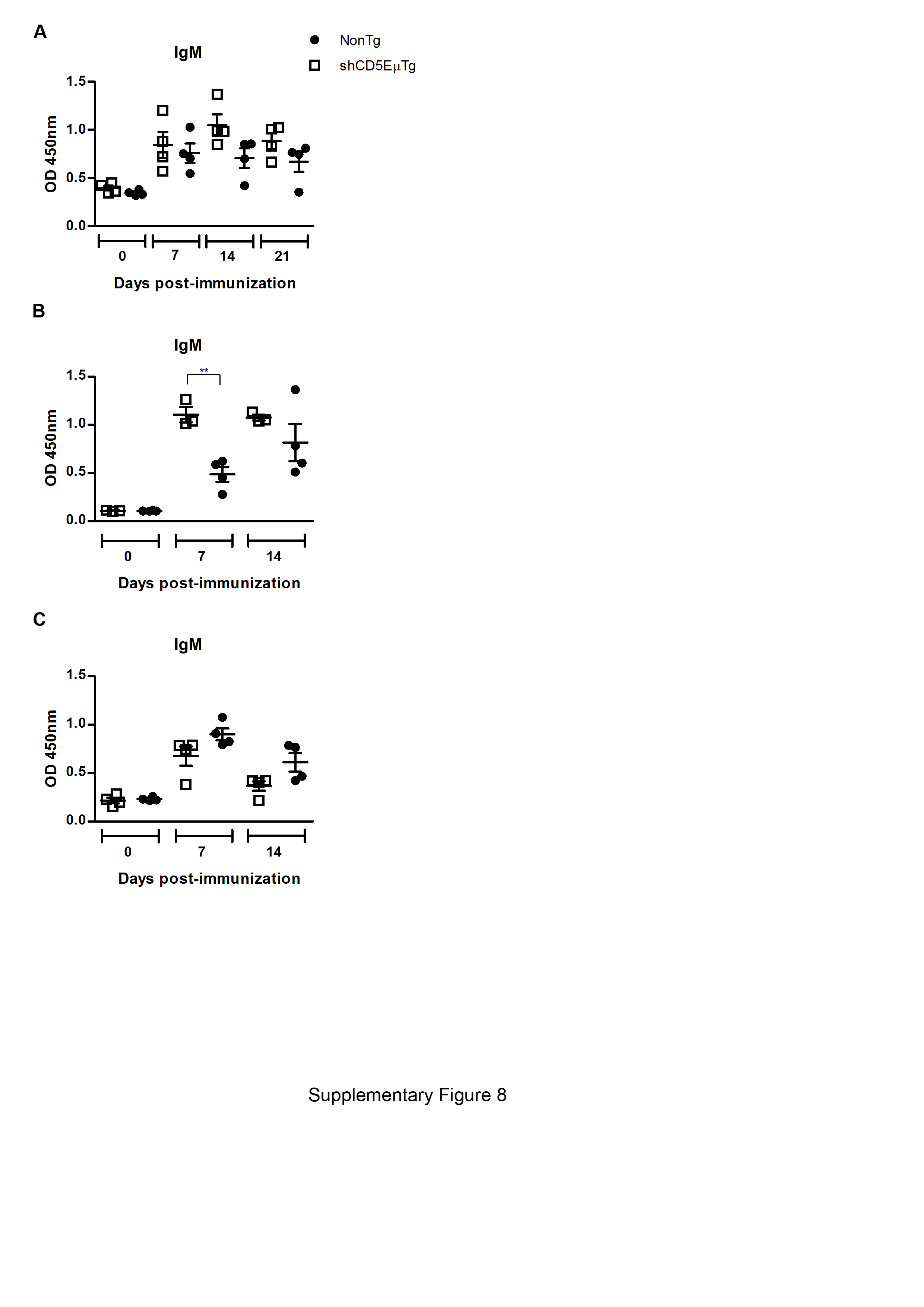

Supplement: Figure S8 — shCD5EμTg and non-transgenic mice IgM response to immunization with T-dependent and T-independent (type 1 and type 2) antigens. Mice were immunized i.p. with 50 µg TNP5-KLH (A), TNP0.3-LPS (B) or TNP65-Ficoll (C) in 200 µl PBS, as examples of TD, TI type 1 and TI type 2 antigens, respectively. Sera from immunized mice were collected at days 0, 7 and 14 (for TI response) or 0, 7, 14 and 21 (for TD response) after the primary immunization and levels of TNP-specific antibodies determined by ELISA. IgM levels are expressed as OD 450 nm values. The bars represent the average value for each group. ** p<0,05. (TIF) [file pone.0084895.s008.tif]

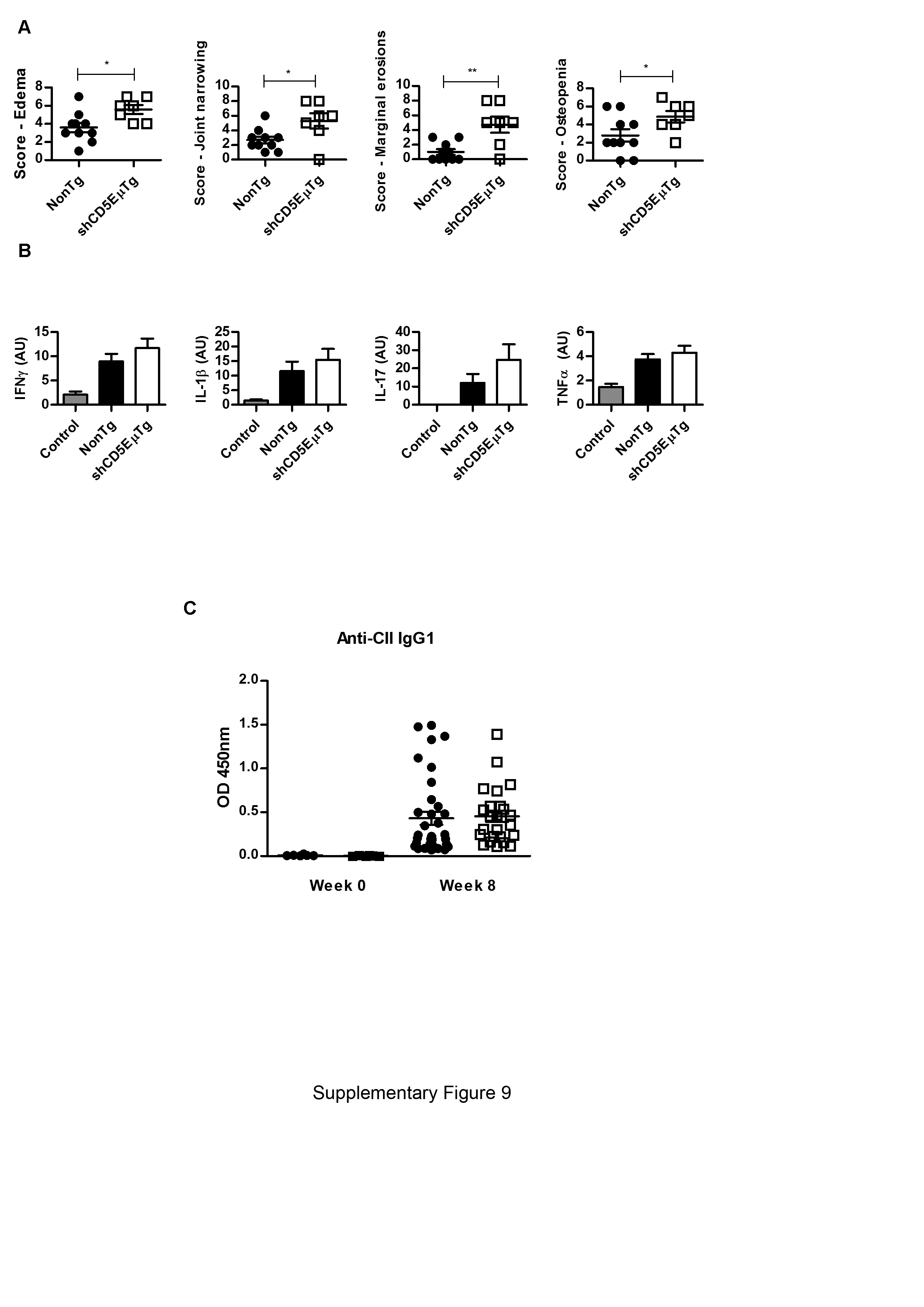

Supplement: Figure S9 — Exacerbated autoimmune disease in shCD5EμTg mice. A) Radiological signs (edema, joint narrowing, marginal erosions and osteopenia) were scored at week 8 for each mouse as described in the Materials and Methods section. B) Cytokine mRNA levels were determined by real time RT-PCR in joint tissue, normalizing results to GAPDH expression levels. Results are expressed in arbitrary units (A.U.). C) IgG1 antibodies against type II collagen were measured by ELISA in the sera of (DBA_B6)F1×shCD5EμTg transgenic mice and non-transgenic littermates at weeks 0 and 8. Results are expressed as OD 450 nm. (TIF) [file pone.0084895.s009.tif]
